# Lacustrine sedimentation by powerful storm waves in Gale crater and its implications for a warming episode on Mars

**DOI:** 10.1038/s41598-023-45068-5

**Published:** 2023-10-31

**Authors:** Ezat Heydari, Jeffrey F. Schroeder, Fred J. Calef, Timothy J. Parker, Alberto G. Fairén

**Affiliations:** 1 Department of Physics, Atmospheric Sciences, and Geoscience, Jackson State University, 1400 Lynch Street, Jackson, MS 39217 USA; 2 Jet Propulsion Laboratory, California Institute of Technology, 4800 Oak Grove Drive, Pasadena, CA 91109 USA; 3 Centro de Astrobiología (CSIC-INTA), Madrid, Spain; 4 Department of Astronomy, Cornell University, Ithaca, NY 14853 USA

**Keywords:** Hydrology, Plant sciences, Climate sciences, Environmental sciences, Planetary science

## Abstract

This investigation documents that the Rugged Terrain Unit, the Stimson formation, and the Greenheugh sandstone were deposited in a 1200 m-deep lake that formed after the emergence of Mt. Sharp in Gale crater, Mars, nearly 4 billion years ago. In fact, the Curiosity rover traversed on a surface that once was the bottom of this lake and systematically examined the strata that were deposited in its deepest waters on the crater floor to layers that formed along its shoreline on Mt. Sharp. This provided a rare opportunity to document the evolution of one aqueous episode from its inception to its desiccation and to determine the warming mechanism that caused it. Deep water lacustrine siltstones directly overlie conglomerates that were deposited by mega floods on the crater floor. This indicates that the inception phase of the lake was sudden and took place when flood waters poured into the crater. The lake expanded quickly and its shoreline moved up the slope of Mt. Sharp during the lake-level rise phase and deposited a layer of sandstone with large cross beds under the influence of powerful storm waves. The lake-level highstand phase was dominated by strong bottom currents that transported sediments downhill and deposited one of the most distinctive sedimentological features in Gale crater: a layer of sandstone with a 3 km-long field of meter-high subaqueous antidunes (the Washboard) on Mt. Sharp. Bottom current continued downhill and deposited sandstone and siltstone on the foothills of Mt. Sharp and on the crater floor, respectively. The lake-level fall phase caused major erosion of lacustrine strata that resulted in their patchy distribution on Mt. Sharp. Eroded sediments were then transported to deep waters by gravity flows and were re-deposited as conglomerate and sandstone in subaqueous channels and in debris flow fans. The desiccation phase took place in calm waters of the lake. The aqueous episode we investigated was vigorous but short-lived. Its characteristics as determined by our sedimentological study matches those predicted by an asteroid impact. This suggests that the heat generated by an impact transformed Mars into a warm, wet, and turbulent planet. It resulted in planet-wide torrential rain, giant floods on land, powerful storms in the atmosphere, and strong waves in lakes. The absence of age dates prevents the determination of how long the lake existed. Speculative rates of lake-level change suggest that the lake could have lasted for a period ranging from 16 to 240 Ky.

## Introduction

**Figure 1:**
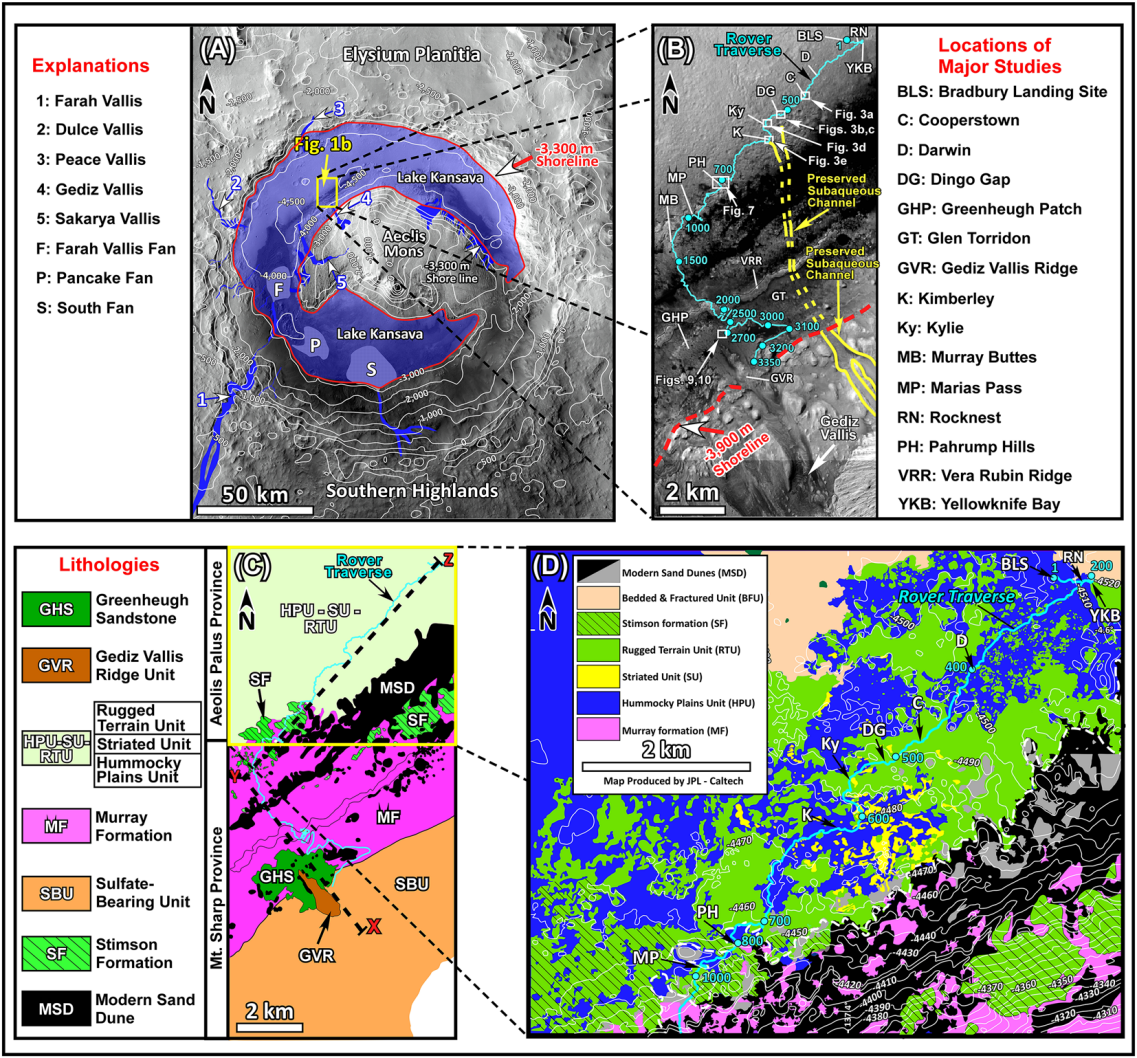
(**A**) Map shows physiographic features of Gale crater and the aerial extent of Lake Kansava at its highstand. White lines are topographic contours at 500 m intervals. Major paleo-channels are from^16^: Farah Vallis (1), Dulce Vallis (2), Peace Vallis (3), Gediz Vallis (4), and Sakarya Vallis (5). Major fans are from^16^: Farah Vallis Fan (F), Pancake Fan (P), and South Fan (S). This map and the one in Fig. 1B were generated from the High Resolution Imaging Science Experiment (HiRISE) base map for Mars Science Laboratory (https://bit.ly/MSL_Basemap). Credit: Calef and Parker^17^. (**B**) Map shows physiographic features of the study area, locations of major campaigns by the Curiosity rover, and areas where images of this report were acquired. One of the subaqueous channels that extended from Mt. Sharp to the Kimberley location is marked by solid and dashed yellow lines. (**C**) Geological map shows distribution of rock units in the study area. The black dashed line is the location of the XYZ profile shown in Fig. 2. (**D**) Geological map shows rock units exposed along the rover’s traverse on the northern part of the crater floor or Aeolis Palus (see Fig. 1C for the location). Here, the RTU is always associated with and consistently overlies the HPU and/or the SU. White lines are topographic contour lines at 10 m intervals. Cyan line, solid cyan circles, and cyan numbers in 1B and D are rover traverse, Sol locations, and Sol numbers, respectively. One Sol is one Martian day. This map is modified from a figure in: Deposits from giant floods in Gale crater and their implications for the climate of early Mars^8^. Credit: Heydari et al.^8^.

Today’s Mars is cold and dry with temperatures averaging about − 60 °C^1–3^. However, geological evaluation of Mars^4^, river channels^5^, in-situ discoveries of fluvial deposition^6–8^, formation of valley networks ^9,10^, and crater degradation^11^ indicate that early Mars was warm enough, at least periodically, for liquid water to flow on its surface, to accumulate in its lakes, and to drain into its possible oceans. In situ examinations of lacustrine strata that were deposited in over 400 paleolakes^12,13^ can provide insights into geological processes of the red planet similar to the study of such deposits on Earth^14^. The landing of the Curiosity rover in one of these paleolakes in Gale crater^15^ (Fig. 1A) provided this opportunity.

Gale is an impact crater that is located near the Martian equator^18^. The crater was completely filled with sediments soon after it formed^4^. However, its margins were later excavated^4^ which created the modern two morphologies of the crater: Aeolis Mons and the crater floor (Fig. 1A). Aeolis Mons, also known as Mt. Sharp, is a crescent shaped mound (Fig. 1A) near the center of the crater and consists of about 5 km of sedimentary rocks^4^. The crater floor surrounds Mt. Sharp (Fig. 1A). The Curiosity rover has been examining the northern part of the crater floor, or Aeolis Palus, and the adjacent slope of Mt. Sharp for the past 11 years^15^ (Fig. 1B,C). The proposed age of Gale crater ranges from the Early Noachian to the Early Hesperian depending on the method used^4,7,11,19,20^. However, geological consideration^4^, stratigraphic constraints^8^, and crater count age dates^19,20^ suggest that the formation of Gale crater, its filling with sediments, and the subsequent erosion of its margins occurred during the Middle to Late Noachian Periods.

**Figure 2:**
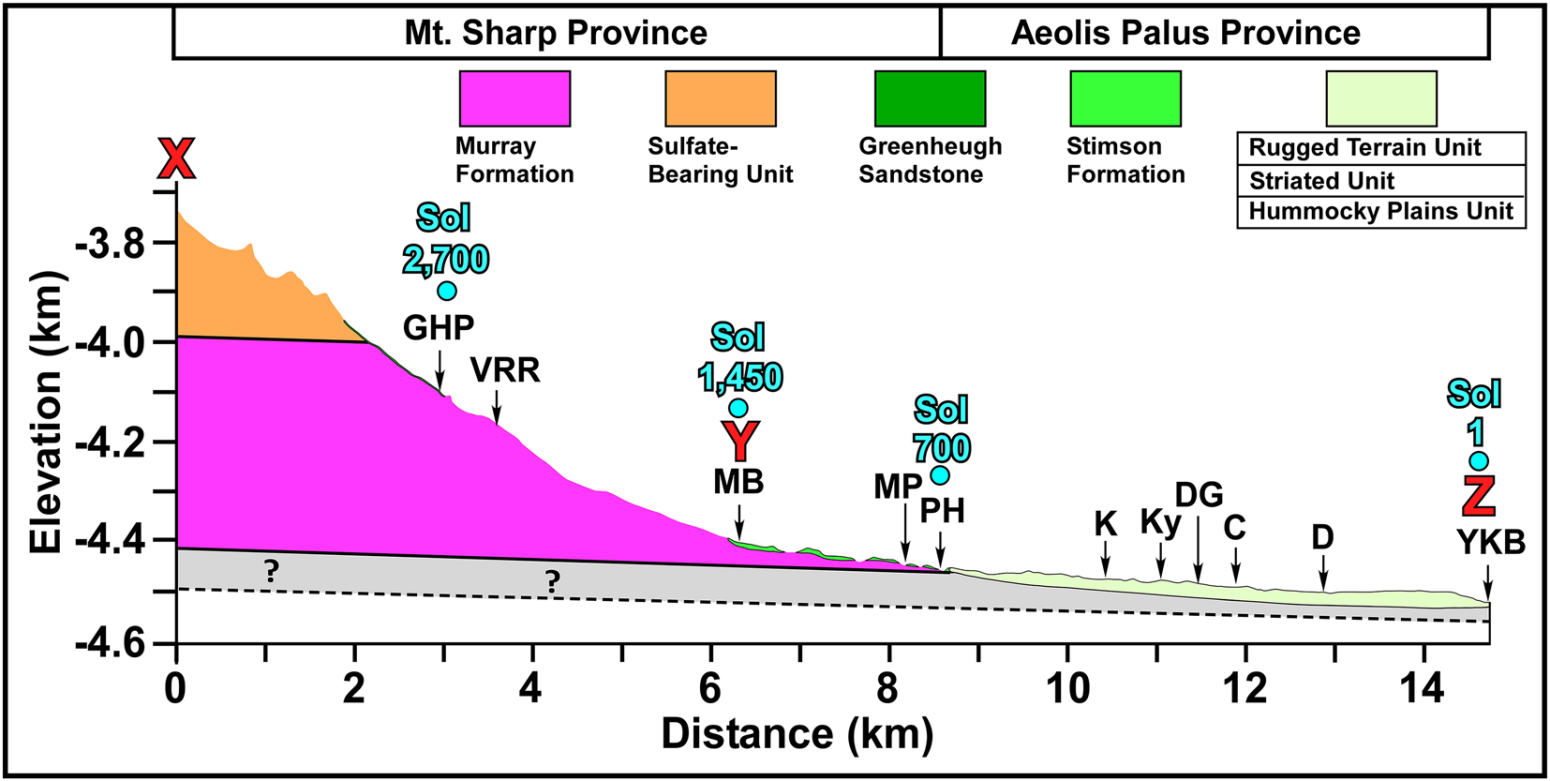
Topographic and geological profile shows rock types that were examined by the Curiosity rover along its traverse. The location of the profile is shown in Fig. 1C. Abbreviations are as those in Fig. 1B.

The goal of this investigation is to present sedimentological and depositional environments of the Rugged Terrain Unit (RTU), the Stimson formation (SF), and the Greenheugh sandstone (GHS) in Gale crater (Figs. 1C,D, 2). They make up about 30 m of strata that occur on the erosion surface of the northern flank of Mt. Sharp (Figs. 1C, 2). We show that these three rock units were deposited in a 1200 m-deep lake that formed after Mt. Sharp was in place and Gale crater had acquired its modern morphology (Figs. 1A, 2). The study documents the evolution of one aqueous episode from its inception to its desiccation and reveals that it had five distinct phases.

The inception phase was sudden and catastrophic when gigantic floods poured into Gale crater. The lake-level rise phase was dominated by shoreline sedimentation under the influence of powerful storm waves. The lake-level highstand phase included sedimentation by strong bottom currents. The lake-level fall phase eroded lacustrine strata on Mt. Sharp and redeposited them on the crater floor as debris flows. The desiccation phase took place in calm waters of the lake.

Our observations suggest that the aqueous episode we investigated was a vigorous but short-lived event. It occurred under warm and wet conditions during the Late Noachian time. The event took place when Mars experienced a global warming event that was triggered by the heat generated by an asteroid impact.

## Background

### Previous studies

The Curiosity rover has provided remarkable insights into geological processes of the modern and the ancient Mars over its past 11 years of exploring Gale crater (Fig. 1). Major discoveries began at the landing site (Fig. 1B). Here, a sedimentological study revealed that conglomerate and sandstone layers of the Hummocky Plains Unit (HPU) were deposited by fluvial processes^6^. This discovery was the first in-situ evidence for running water on Mars indicating that the red planet was warm enough for liquid water to be stable on its surface in the past, an idea that has been suspected for several decades^1–5^.

New discoveries continued when the rover conducted its first investigation of a modern aeolian sand deposits at the Rocknest location (Fig. 1B,D) during Sol 59 to Sol 100, where one Sol is one Martian day. Investigations at this location showed that the modern aeolian sand in Gale crater consists primarily of olivine, pyroxene, plagioclase, and amorphous materials^21,22^, all highly unstable components that can be easily removed during chemical weathering^23^. This is unlike Earth where similar deposits are composed primarily of one mineral: quartz^24^.

Perhaps the most important finding was made at the Yellowknife Bay (YKB) area, about 70 m to the east of the Rocknest location (Fig. 1B,D). The study of diverse lithologies exposed at this location documented deposition in a fluvio-lacustrine environment indicating that habitable conditions existed on Mars early in its history^25^. Surprisingly, however, the mineralogy and elemental compositions of nearly 4 billion-year old strata at the YKB area are similar to the modern aeolian sand of the Rocknest region^26,27^ indicating minimal chemical weathering on Mars in the past^28^.

The rover encountered three distinctive rock units with extensive exposures along its ascent route from the YKB area to Mt. Sharp (Fig. 1C,D). They are: the Striated Unit (SU), the Rugged Terrain Unit (RTU), and the HPU^7,25,29^. These rock units are lithologically distinct, display specific morphological occurrences^7,25,29^, and occur in a well-defined stratigraphic order throughout their exposures^8^. The HPU always occurs at the base (Fig. 1D) and consists of cobble to boulder conglomerate that forms smooth surface hummocks. The SU consists of numerous patches of south-dipping beds that overlie hummocks of the HPU^7,8,25,29^ (Fig. 1D). The RTU forms a blanket of strata with rugged cliffs that overlie both the HPU and the SU^30–32^ (Fig. 1D).

The rover reached the Pahrump Hills location at the foothill of Mt. Sharp over two Earth years after its landing in Gale crater (Fig. 1B–D). Surprisingly, the three rock units with extensive exposures on Aeolis Palus (the HPU, the SU, the RTU) were absent at this new location (Fig. 1C,D). Instead, the rover encountered two new lithologies (Fig. 1C,D): the Murray formation (MF) and the Stimson formation (SF).

The MF is only 13 m thick at Pahrump Hills ^7,33–35^ but its exposures extend uninterrupted uphill to the Vera Rubin Ridge and the Glen Torridon regions indicating that it is over 500 m thick (Figs. 1C,D, 2). Lithologically, the MF consists primarily of thin-bedded and laminated mudstone that is interpreted as lacustrine^7,33–37^. Recently, however, it has been suggested that the MF transitions upward into strata of aeolian and fluvial origin^38–41^. The SF is an unsorted, cross-bedded coarse-grained sandstone that overlies the MF with a sharp contact^42^. The SF has been interpreted as an aeolian erg deposit^42^.

The rover spent two Earth years along the northern foothill of Mt. Sharp examining characteristics of the SF and the MF from Pahrump Hills to Murray Buttes (Fig. 1B–D). Afterwards, it began its one Earth-year climb over the slopes of Mt. Sharp toward a prominent geomorphic feature: the Vera Rubin ridge (Fig. 1B–D). Detailed examinations demonstrated that the ridge actually consists of the MF that experienced diagenetic overprints^43^.

Then, the rover entered the Glen Torridon region, an elongated area with low elevations south of the Vera Rubin ridge (Fig. 1B). Curiosity spent two Earth years there and conducted detailed investigations of an interval which had been previously named the Clay-bearing Unit^7^. Studies suggested that lacustrine mudstones of the MF transition into fluvial deposits in the Glen Torridon region^39–41^.

While at Glen Torridon, the rover also performed a detailed study of a 2 m-thick sandstone that forms a distinct 2.5 km × 2.5 km morphological feature: the Greenheugh patch (Fig. 1B,C). The sandstone that forms the patch is here named the Greenheugh sandstone: GHS (Fig. 1C).

After the completion of the campaign at Glen Torridon, the rover continued its two Earth-year ascent of Mt. Sharp examining strata along the way (Fig. 1B,C). The rover currently studies the Sulfate-Bearing Unit (SBU) and results are being analyzed by the MSL science team (Figs. 1B,C, 2). Major discoveries have already been made about the mineralogy, elemental compositions, and isotopic signatures of the SBU. The study of a prominent feature called the Marker Band^44,45^ is one such case.

The above background summary emphasized investigations with direct implications to the main focus of our study. Additional scientific results about MSL research in Gale crater include significant contributions on mineralogy^46^, bulk elemental composition^47–49^, elemental analysis by laser ablation^50–52^, diagenesis^53–55^, isotopic compositions^56,57^, organic geochemistry^58–63^, atmospheric and aeolian processes^64–66^, and regional geological and sedimentological investigations^43,67–69^.

### Deposition of strata visited by the rover in Gale crater

There are currently two viewpoints about the deposition of strata that have been visited by the Curiosity rover so far. The first is a one lake model^7^. This interpretation suggests that all strata that occur on Aeolis Palus (the HPU, the SU, the RTU) and those that are exposed at lower elevations of Mt. Sharp (the MF), except the SF and the GHS, were deposited in a fluvio-deltaic-lacustrine system^7^. The body of water in which the lacustrine strata of this model were deposited is Gale lake^7^. It existed prior to the emergence of Mt. Sharp^7^. After Gale lake dried up, the fluvial-deltaic-lacustrine strata were buried by about 4 km of strata. They were subsequently re-exposed when margins of Gale crater were excavated and Mt. Sharp emerged. These events were followed by a cold and dry period during the Hesperian which led to the formation of a large aeolian erg and deposition of the SF and the GHS^42,70^.

A new perspective on deposition of strata that have been examined by the rover has recently emerged^8,16,71^. Sedimentological observations^8,71^ and geomorphic evidence^16^ suggest the presence of at least two lakes in Gale crater: Gale Lake^7^ and Lake Kansava (this study). Gale lake existed when Mt. Sharp had not yet emerged^7^. The MF that is now exposed on the slopes of Mt. Sharp (Fig. 2) was deposited in Gale lake. Lake Kansava (Fig. 1A) formed after the formation of Mt. Sharp^8,71^. The RTU, the SF, and the GHS were deposited in Lake Kansava (Fig. 1A).

## New observations

This investigation provides new insights into lithology, sedimentology, and depositional environment of the Rugged Terrain Unit (RTU), the Stimson formation (SF), and the Greenheugh sandstone (GHS). Each rock unit occupies a distinct geography in Gale crater (Fig. 1C). The RTU occurs exclusively on Aeolis Palus or areas with the lowest elevations in Gale crater (Figs. 1C,D, 2). The SF is restricted to a 2 km band along the northern foothills of Mt. Sharp (Figs. 1C,D, 2). The GHS forms a 2.5 × 2.5 km exposure, the Greenheugh patch, on the slope of Mt. Sharp (Fig. 2). The SF and the GHS were deposited on the erosion surface of the northern slopes of Mt. Sharp (Fig. 2) suggesting that they formed after Gale crater had acquired its modern morphology^8^. The RTU overlies conglomerates and sandstones of the HPU and the SU whose deposition also post dates the emergence of Mt. Sharp^8^ (Fig. 2).

### The Rugged Terrain Unit (RTU)

**Figure 3:**
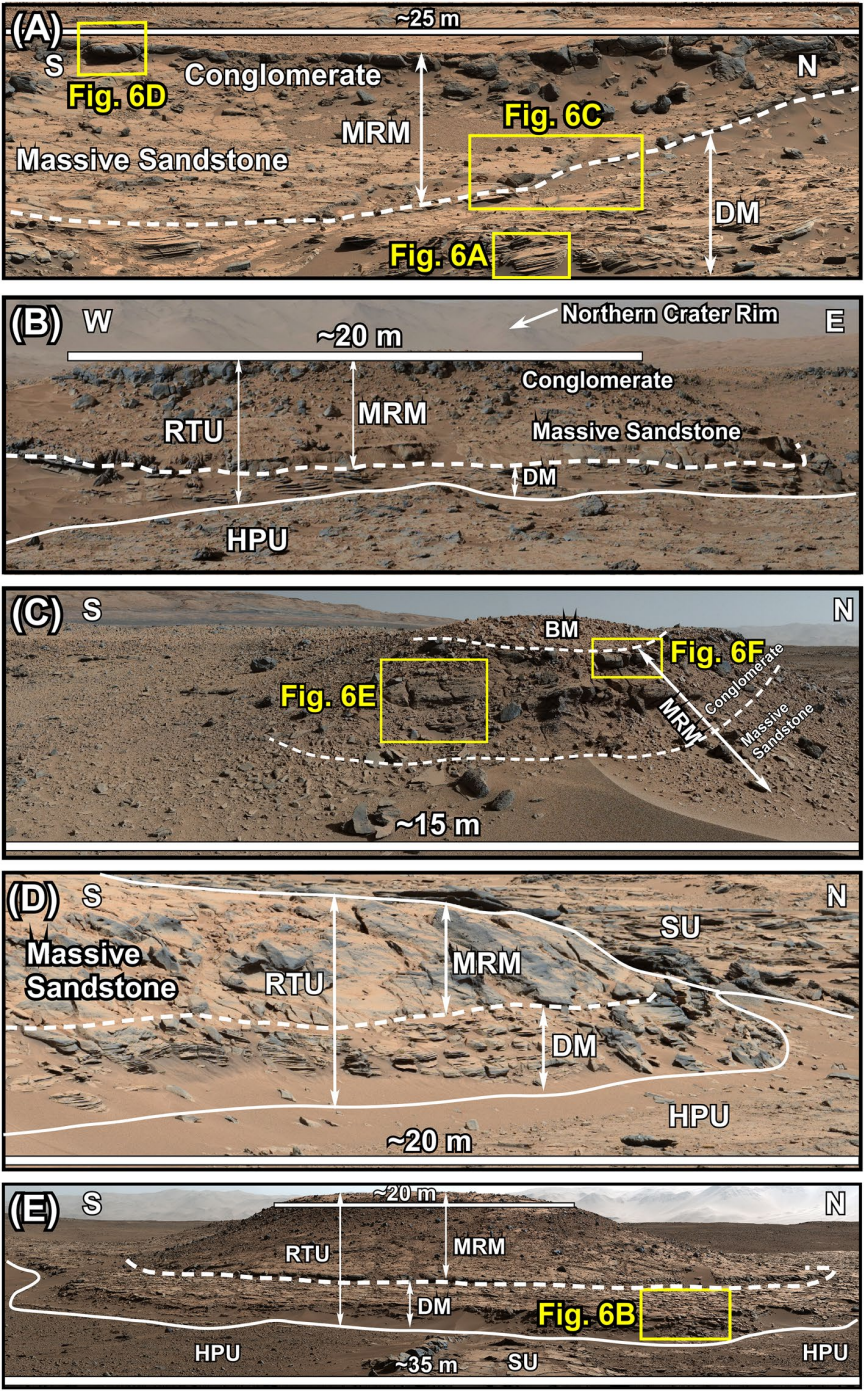
Mastcam images show characteristics of the Rugged Terrain Unit (RTU) at the Cooperstown (**A**), the Dingo Gap (**B**), the South Wall of the Dingo Gap (**C**), the Kylie (**D**), and the Kimberley (**E**) locations. The RTU overlies the Hummocky Plains Unit (HPU) and/or the Striated Unit (SU) with sharp contacts at all locations. The Dillinger member (DM) consists of thin-bedded siltstone to fine-grained sandstone. It is about 2 m thick at the Kimberley location but thins northward to about 0.5 m at the Cooperstown area (see Fig. 4). The Mt. Remarkable member (MRM) is about 3 m thick at the Kimberley area but thins northward to less than 1 m at the Cooperstown location (Fig. 4). The lithology and the geometry of the MRM changes from north to south. At the Cooperstown, the Dingo Gap, and the Kylie locations, the MRM forms a near horizontal layer and is composed of a massively-bedded sandstone that grades upward to a massively-bedded, matrix supported conglomerate. At the Kimberley location, however, the MRM occurs as a north–south oriented ridge and consists of a basal conglomerate that grades upward to a sandstone and shows fining upward grain size distribution (see Fig. 4). The contact between the MRM and the DM is sharp and erosional at all localities. The BM consists of about 1 m sandstone and siltstones. It is cross bedded at the base but becoms of laminated toward the top. It overlies the massive conglomerate of the MRM with a sharp contact. It is best preserved at the Dingo Gap (Mastcam mosaic C). Unprocessed images used to generate mosaics of this figure are publically available at the Planetary Data System web site at https://pds-imaging.jpl.nasa.gov/. The credit for the Mastcam mosaic images of this figure goes to Malin Space Science Systems and NASA/JPL. Please see the Supplemental Document 1 for additional information on these images.

**Figure 4:**
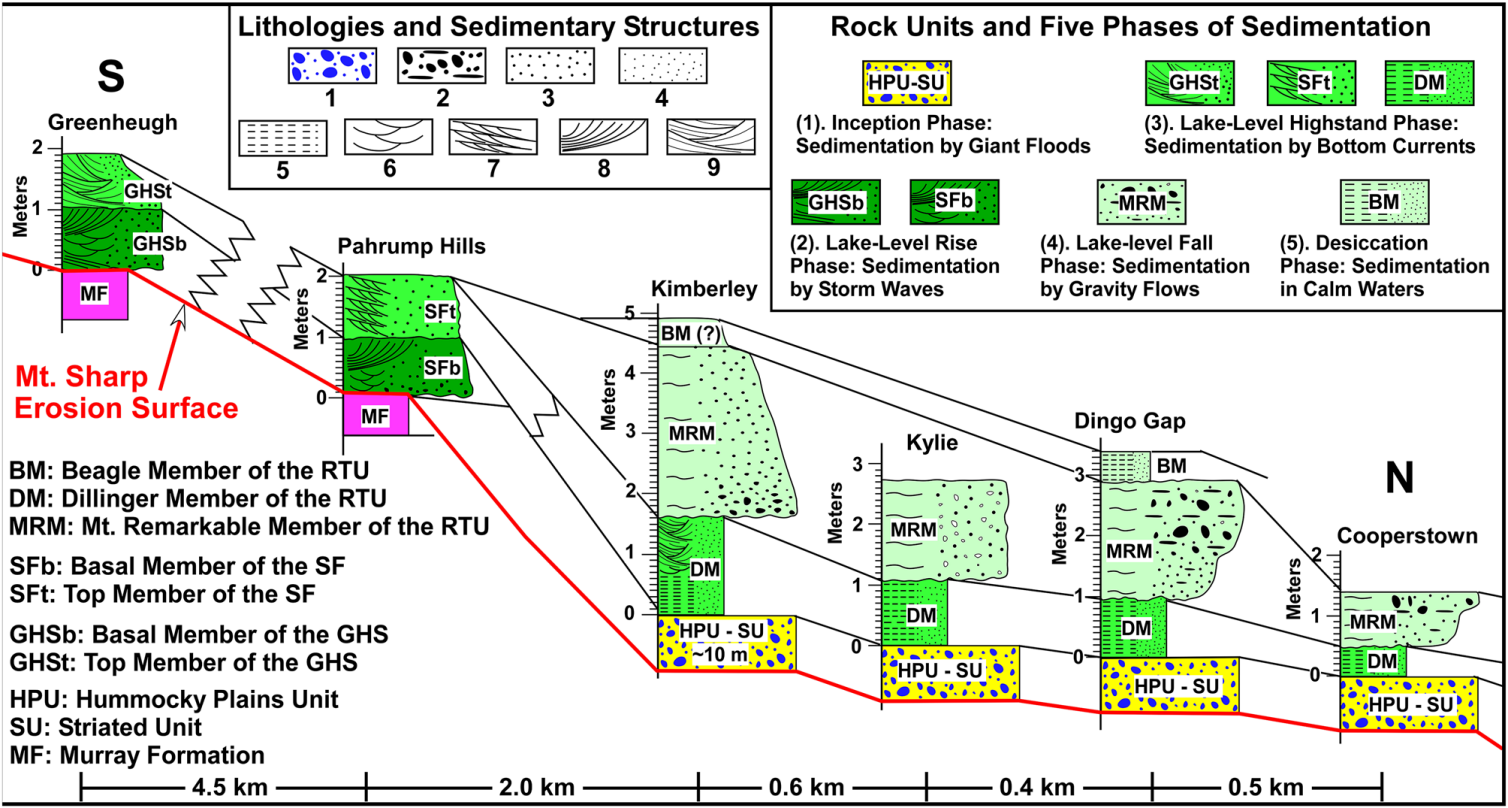
The diagram shows a north—south stratigraphic correlation of the RTU, the SF, and the GHS along the rover's traverse path. These three rock units were deposited in Lake Kansava over the erosion surface of the northern flank of Mt. Sharp (see Fig. 2). The graph also shows rock units that formed during the 5 phases of the aqueous episode we studied. They are as follow in the chronological order from the oldest to the youngest. (1) The HPU and the SU were deposited by giant floods during the inception phase of the aqueous episode prior to the establishment of the Lake Kansava^8^. (2) The SFb and GHS were deposited during the lake-level rise phase under the influence of powerful storm waves. (3) the GHSt, the SFt, and the DM were deposited during the lake-level highstand phase by strong bottom currents. (4) The MRM was deposited as debris flows during the lake-level fall. (5) The BM was deposited by suspension in calm waters of the lake during the desiccation phase of the lake. Symbols for lithologies and sedimentary structures are: 1: Conglomerate. 2: Conglomerate with flat-pebble orientation. 3: Coarse- to medium-grained sandstone. 4: Fine-grained sandstone to siltstone. 5: Laminations. 6: Trough cross-beds. 7: Cross bed bundles. 8: Large cross beds. 9: Lens-shaped packages of cross bedded layers.

The RTU forms a blanket of near horizontal layers in areas with the lowest elevations on the northern part of the crater floor (Aeolis Palus) in Gale crater (Figs. 1C,D, 2). This rock unit is always associated with and overlies the HPU and/or the SU^8^ (Figs. 1D, 2, 3). The RTU is up to 8 m thick and its outcrops were studied and imaged at the Cooperstown (Fig. 3A), the Dingo Gap (Fig. 3B,C), the Kylie (Fig. 3D), and the Kimberly (Fig. 3E) locations. However, geological mapping shows that its exposures extend to the foothills of Mt. Sharp (Fig. 1D). The RTU consists of three members^30–32^: The Dillinger member (DM) at the base, the Mt. Remarkable member (MRM) in the middle, and the Beagle member (BM) at the top (Figs. 3, 4).

**Figure 5:**
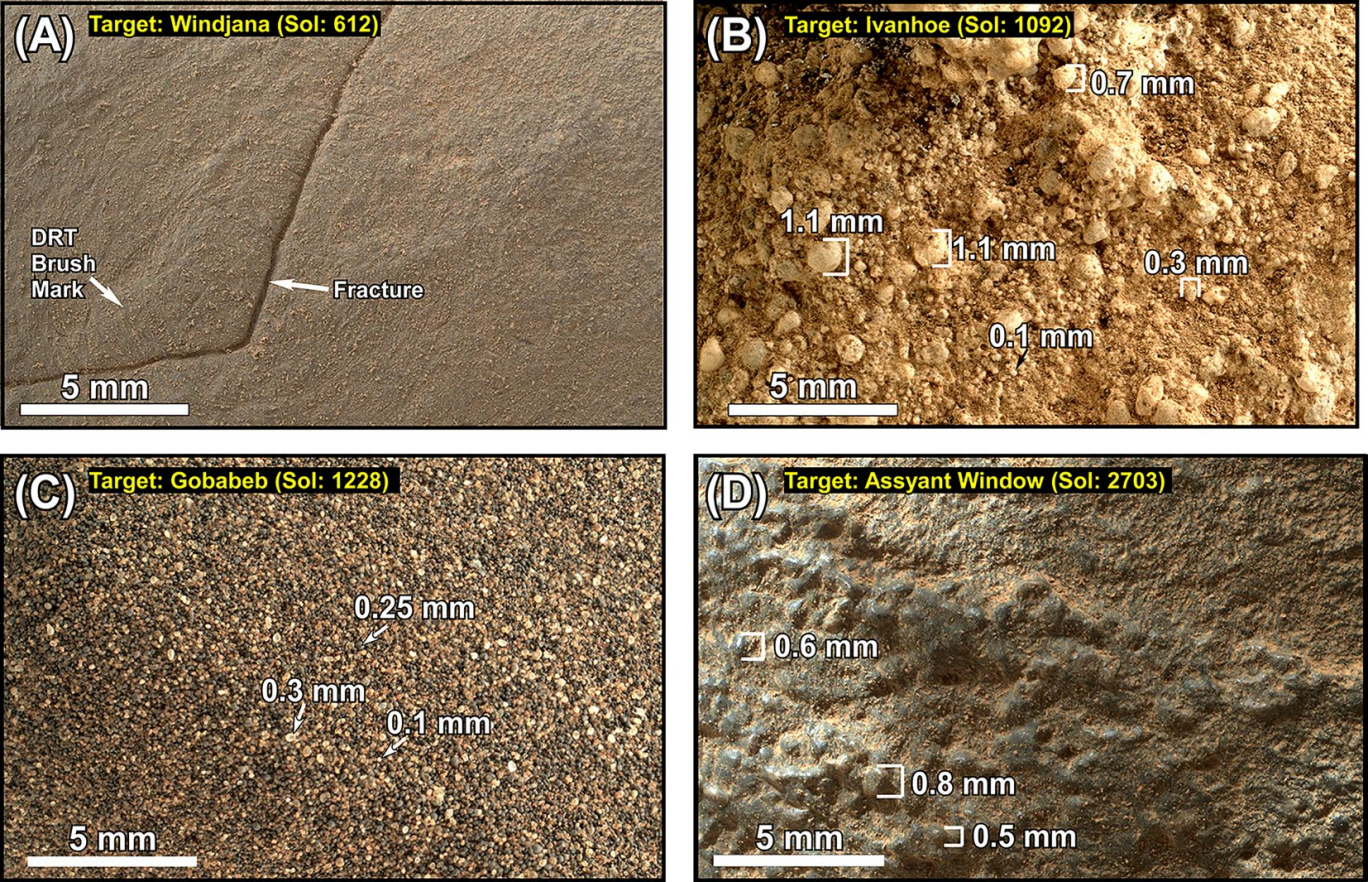
(**A**) Mars Hand Lens Imager (MAHLI) photograph at 1 cm standoff shows that the Dillinger member (DM) of the RTU consists of a siltstone to a very fine-grained sandstone. The surface of the target was brushed by the Dust Removal Tool (DRT) of the rover. (**B**) MAHLI photograph at 2 cm standoff shows that the Stimson formation (SF) consists of a poorly-sorted coarse-grained sandstone. (**C**) MAHLI photography at 1 cm standoff shows that the modern aeolian dunes in Gale crater are composed of well-sorted, fine grained sand. (**D**) MAHLI photography at 1 cm standoff shows that the Greenheugh sandstone (GHS) is medium-grained and moderately to well sorted. Unprocessed images of this figure are publically available at the Planetary Data System web site at https://pds-imaging.jpl.nasa.gov/. The credit for the Mastcam mosaic images of this figure goes to Malin Space Science Systems and NASA/JPL. Please see Supplemental Document 1 for additional information on these images.

**Figure 6:**
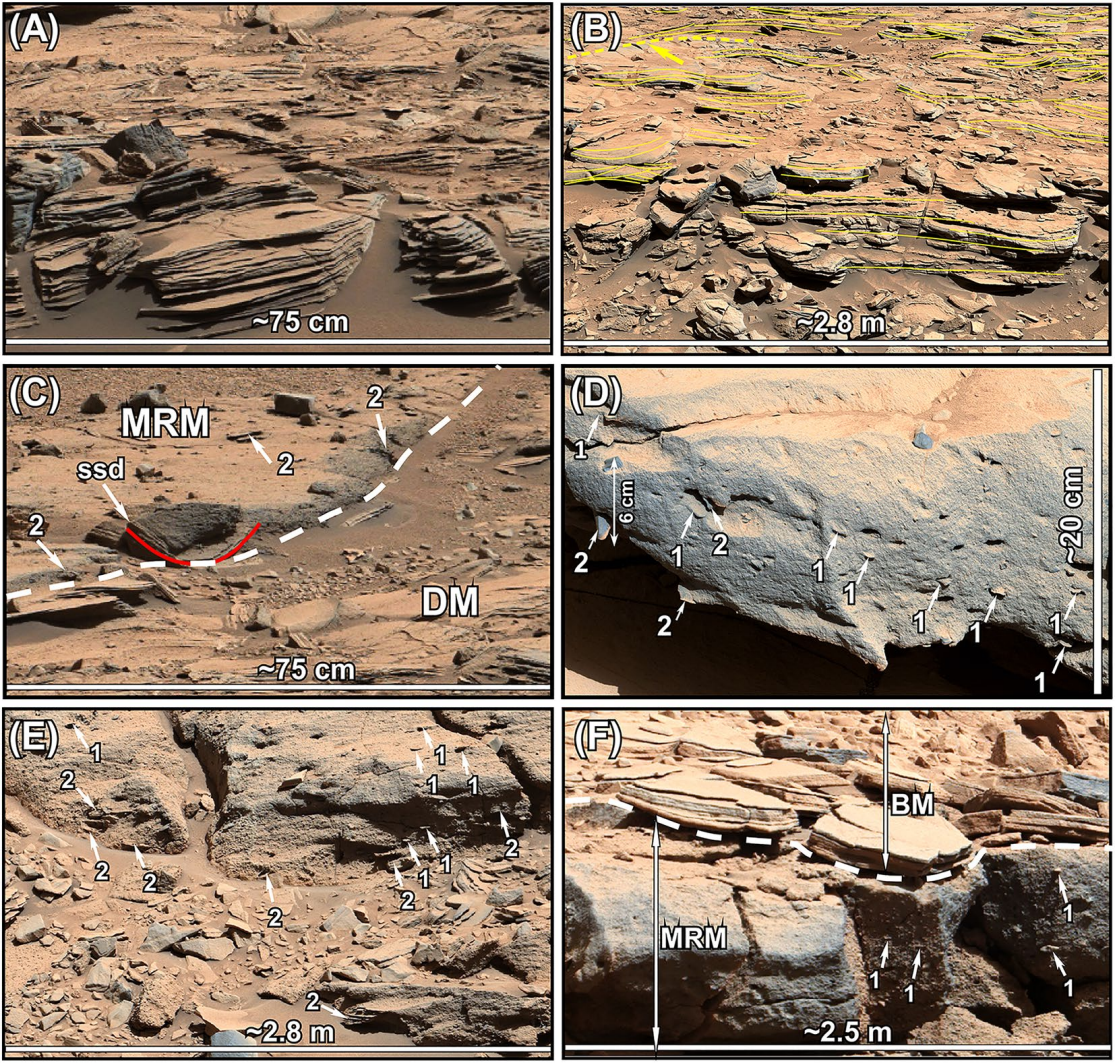
(**A**) The enlarged portion of Fig. 3A shows thin-bedded siltstone to fine-grained sandstone layers of the Dillinger member (DM) at the Cooperstown location. (**B**) Mastcam image mosaic shows abundant bedforms in the DM at the Kimberley location (see Fig. 3E for location). Thin solid yellow lines mark the layering. The thick dashed yellow line marks the crest of one preserved antidune ridge. (**C**) The enlarged portion of Fig. 3A shows the contact (solid dashed white line) between the massive sandstone lithology of the Mt. Remarkable member (MRM) and the underlying DM. ssd: soft-sediment deformation feature that is also marked by a red line. (**D**) Mastcam image mosaic shows the conglomerate lithology of the MRM at the Cooperstown area (see Fig. 3A for location). (**E**) Mastcam image mosaic shows the massively-bedded conglomerate lithology of the MRM at the South Wall of the Dingo Gap location (see Fig. 3C for location). (**F**) Mastcam image mosaic shows the contact between the conglomerate lithology of the MRM and the sandstone lithology of the Beagle member (BM) at the South Wall of the Dingo Gap (see Fig. 3C for location). White arrows 1 in all figures point to flat pebbles with subhorizontal orientation. White arrows 2 point to cobbles and boulders. Unprocessed images used to generate mosaics of this figure are publically available at the Planetary Data System web site at https://pds-imaging.jpl.nasa.gov/. The credit for images of this figure goes to Malin Space Science Systems and NASA/JPL. Please see Supplemental Document 1 for additional information on these images.

The DM consists of thin-bedded siltstone to fine-grained sandstone (Figs. 3A,B,D,E, 5A, 6A,B). It is about 2 m thick at the Kimberley region but thins northward to less than 0.5 m at the Cooperstown location (Figs. 3A,D, 4). However, its lithology remains remarkably uniform (Fig. 3). Thin beds of the DM are laminated,  contain symmetrical ridges (Fig. 6B), and display cross beds that dip both downhill and uphill or northward and southward, respectively (Fig. 6B). Most importantly, thin bedded siltstone layers of the DM overlie conglomerates of the HPU and/or the SU with sharp contacts (Figs. 2, 3A,B,D,E).

The MRM is 1–5 m thick (Figs. 3A–E, 4). At the Cooperstown, the Kylie, and the Dingo Gap locations, the MRM consists of 1–3 m of subhorizontal layers of massively-bedded sandstone that grades upward to 1–3 m of matrix supported massively-bedded conglomerate (Figs. 3A–D, 4, 6D,F). Together they show a coarsening upward grain size distribution (Fig. 4). Flat pebbles in the conglomerate show preferred subhorizontal orientation (Fig. 6D,F). At the Kimberley location, however, the MRM forms a linear north–south oriented ridge that begins with a pebbly sandstone to sandy conglomerate at the base and grades upward to a sandstone (Figs. 3E, 4) forming a fining upward lithology. The MRM overlies siltstone layers of the DM with sharp and truncational contacts at all locations (Fig. 3A,B,D,E). The boundary shows soft sediment deformation (Fig. 6C).

The BM is only preserved at the south wall of the Dingo Gap location (Fig. 3C). It is about 1 m thick (Fig. 3C) and overlies the massive conglomerates of the MRM with a sharp contact (Fig. 6F). The BM was beyond the reach of the rover for close examinations. Observations on Mastcam images (Fig. 6F) indicate that it consists of layers of sandstone to siltstone that are centimeters thick and appear cross-bedded sandstone at the base and laminated at the top (Fig. 6F).

### The Stimson formation (SF)

**Figure 7:**
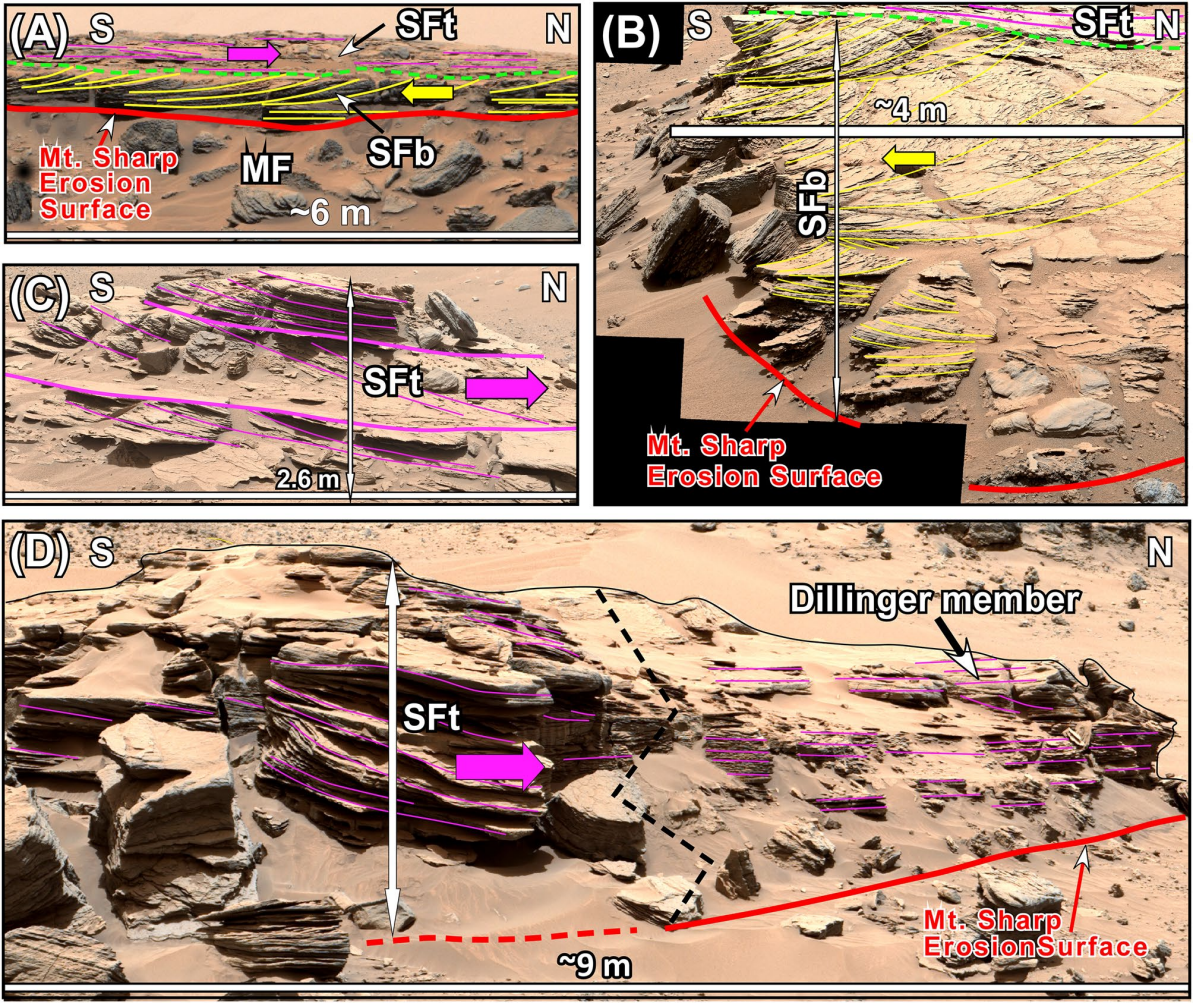
(**A**) A portion of the Mastcam image mosaic shows large cross beds of the basal member of the Stimson formation (SFb) that migrated up the slope of Mt. Sharp or southward. Yellow arrow shows the direction of cross bed migration. The top member of the Stimson formation (SFt) overlies the SFb with a sharp, truncational contact (dashed green line). (**B**) A portion of the mastcam image mosaic shows that the SFb begins with coarse-grained sandstone with trough cross beds with opposing dip angles. This is followed by a layer of sandstone with large trough cross beds that shows flow direction up the slope of Mt. Sharp or southward. Yellow arrow shows the direction of cross bed migration. (**C**) A portion of the mastcam image mosaic shows that the SFt is composed of bundles of cross beds that show flow direction down the slope of Mt. Sharp or northward. Purple arrow shows the direction of migration of cross beds. (**D**) The mastcam image mosaic shows that cross-bedded sandstones of the SFt transition into horizontally layered strata of the Dillinger member (DM). Yellow and purple lines trace layering. Unprocessed images used to generate mosaics of this figure are publically available at the Planetary Data System web site at https://pdsimaging.jpl.nasa.gov/. The credit for mastcam mosaic images of this figure goes to Malin Space Science Systems and NASA/JPL. Please see the Supplemental Document 1 for additional information on these images.

Exposures of the SF are restricted to a 2 km-band along the northern foothills of Mt. Sharp (Fig. 1C,D). This rock unit overlies the mudstones of the MF with a sharp contact (Figs. 4, 7A,B). The SF consists of a cross-bedded, poorly-sorted, coarse-grained sandstone (Fig. 5B) that is 2–4 m thick at the Pahrump Hills location (Fig. 4), but it thickens toward the Murray Buttes region^70^. The SF has two members at Pahrump Hills where it was examined for this study (Figs. 4, 7). The basal member (SFb) consists of sandstones with a prominent 0.5–1 m thick cross bed that clearly shows a flow direction up the slope of Mt. Sharp or southward (Fig. 7A). That is, this cross bed was moving uphill over the slope of Mt. Sharp (Fig. 7A). Occasionally, however, this sandstone layer overlies beds of sandstone with trough cross beds with opposing dip angles (Fig. 7B).

The top member of the SF (SFt) also consists of sandstones. However, it displays distinct bundles of cross beds that show flow direction downhill on the slope of Mt. Sharp or northward (Figs. 4, 7C,D). That is, cross beds of the SFt moved downhill or 180 degrees opposite to the cross beds of the SFb (Figs. 4, 7C,D). Most importantly, the cross bedded sandstones of the SFt transition into thin-bedded horizontal layers that are identical to the DM of the RTU at Pahrump Hills (Fig. 7D).

### The Greenheugh sandstone (GHS)

**Figure 8:**
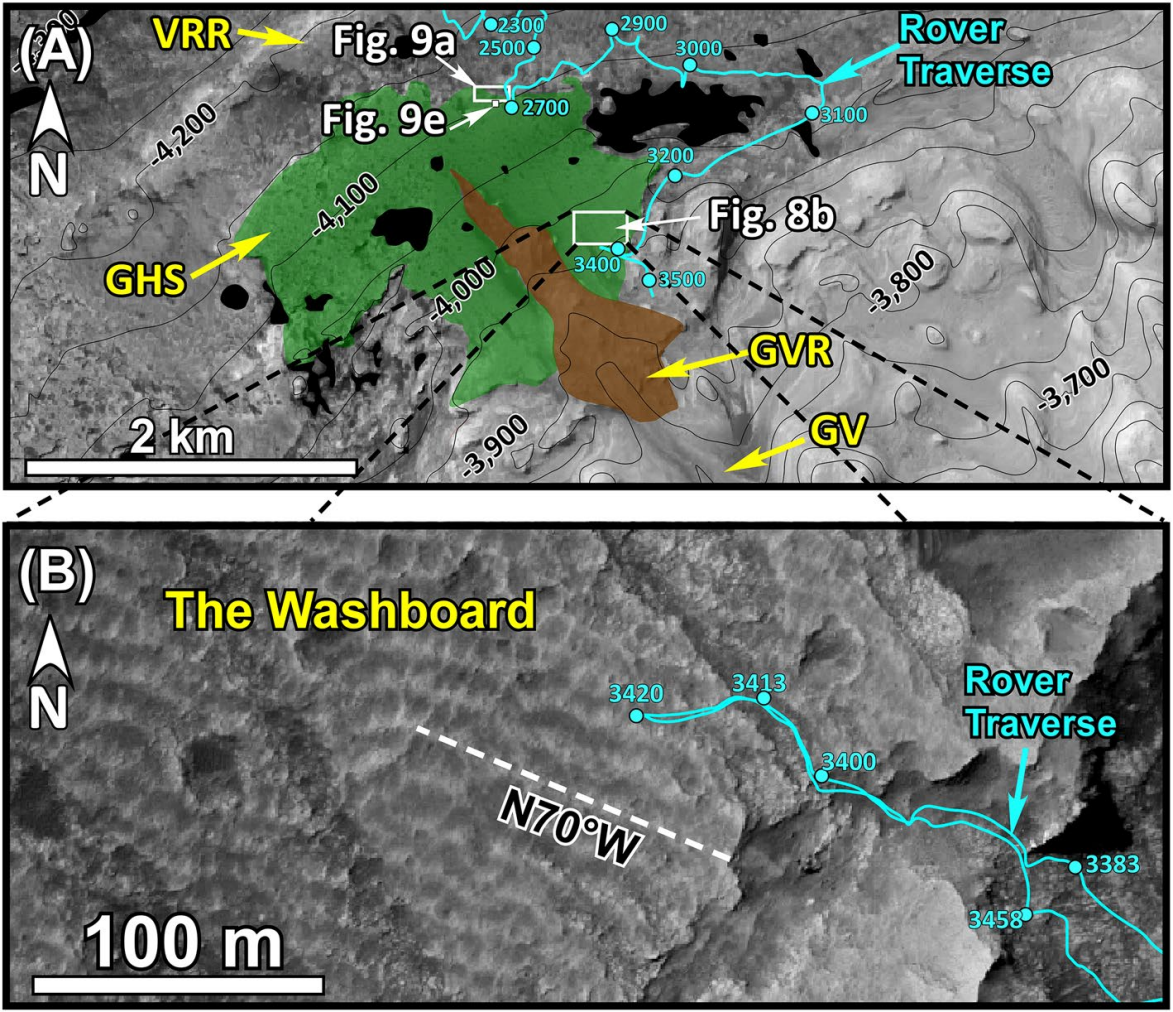
(**A**) Map shows the Greenheugh patch outcrop of the Greenheugh sandstone (GHS). Thin black lines are topographic contour lines at 50 m intervals. Black numbers are elevations in meters. This map was generated from the High Resolution Imaging Science Experiment (HiRISE) base map for Mars Science Laboratory (https://bit.ly/MSL_Basemap). Credit: Calef and Parker^17^. (**B**) An enlarged area of Fig. 8A shows symmetrical antidune ridges, also known as the Washboard^18^, that cover the entire surface of the Greenheugh patch. These ridges are about 100 m long, 0.5 m to 1 m high, have a spacing of 15 m, and are oriented at about N70ºW. Cyan line, cyan circles, and cyan numbers are the rover traverse, Sol locations, and the Sol numbers, respectively. White dashed line marks the crest of one antidune ridge.

**Figure 9:**
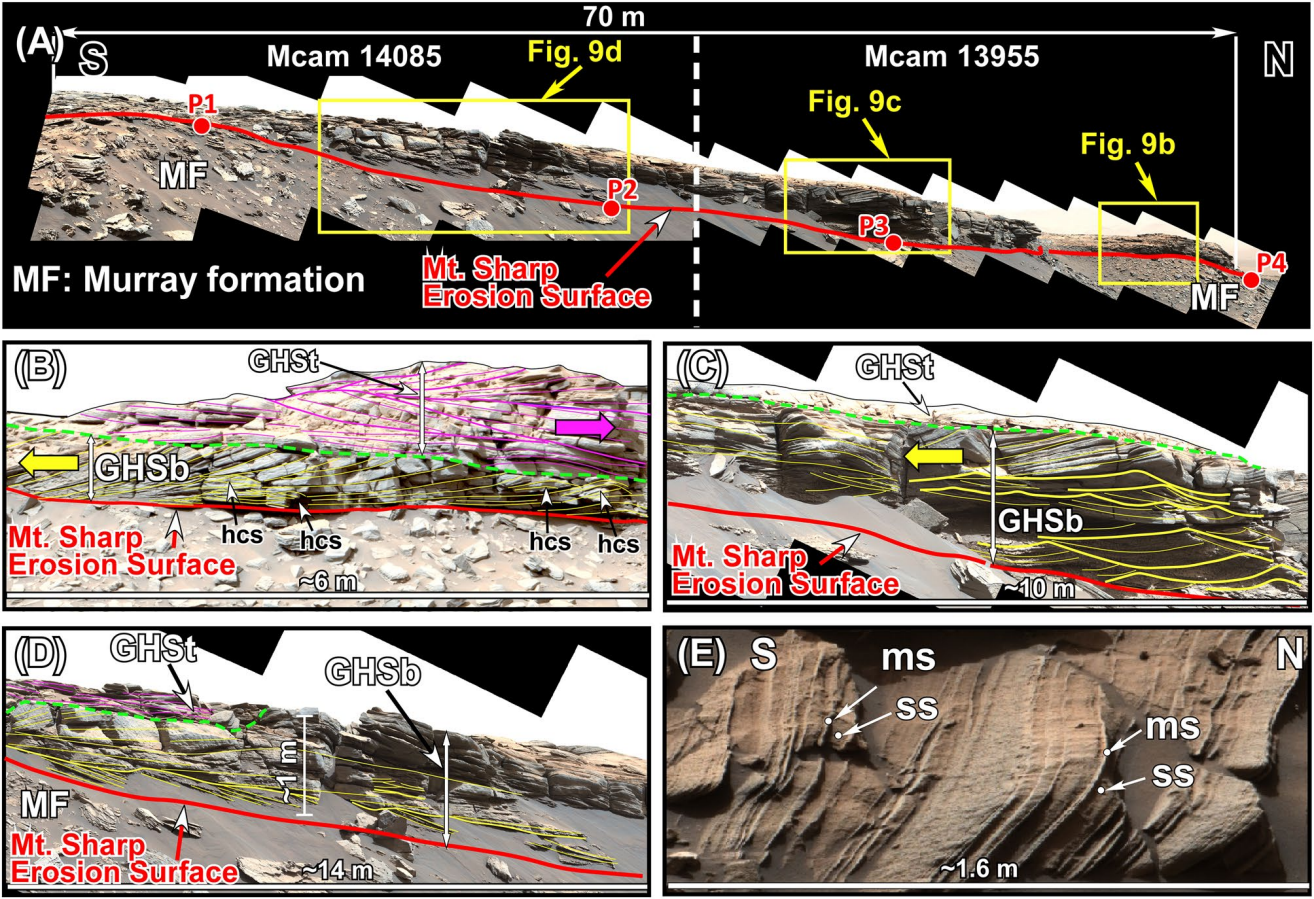
(**A**) Mastcam image mosaics show lithologic and sedimentological characteristics of the GHS along a 70 m-long outcrop at the northern edge of the Greenheugh patch (see Fig. 8A for the location). (**B**–**D**) Mastcam image mosaics show characteristics of the basal (GHSb) and the top (GHSt) members of the GHS at the northern end, the middle, and at the southern end, respectively, of the outcrop in Fig. 9A. hcs: hummocky cross stratification. (**E**) Part of the Mastcam image mosaic shows the top, eroded surface (map view) of the foreset beds of the large cross bed that forms the GHSb (see Fig. 8A for the location). ss: sandstone; ms: mudstone. Yellow and purple lines trace layering. Large yellow and purple arrows show flow direction of the migration of cross beds. Thick yellow lines in (**C**) trace symmetrical ripples. Unprocessed images used to generate mosaics of this figure are publically available at the Planetary Data System web site at https://pds-imaging.jpl.nasa.gov/. The credit for Mastcam mosaic images of this figure goes to Malin Space Science Systems and NASA/JPL. Please see Supplemental Document 1 for additional information on these images.

The GHS occurs as a large outcrop patch (Greenheugh patch) on the slope of Mt. Sharp (Fig. 8), directly uphill from the area where the SF is exposed (Fig. 1B, C), but the two rock units are not physically connected (Figs. 1B,C, 2). The GHS also overlies mudstone lithologies of the MF with a sharp contact (Figs. 2, 4, 9A–C). The GHS has been considered to be the uphill extension of the SF by previous researchers^70^. Its specific morphological exposure as a patch on the slope of Mt. Sharp, its sedimentological characteristics, and to prevent confusion prompted us to informally name it the GHS.

The GHS was examined along a 70 m-long exposure on the northern edge of the Greenheugh patch (Fig. 9A). Here, the GHS consists of a basal and a top member: the GHSb and the GHSt, respectively (Figs. 4, 9B–D). Both members consist of moderate to well sorted medium-grained sandstone (Fig. 5D).

Sedimentological characteristics of the GHSb changes along the 70 m-long outcrop (Fig. 9A). At its northernmost end (downhill), the GHSb is composed of a sandstone with a single, meter-thick cross bed that shows flow direction up the slope of Mt. Sharp or southward (Fig. 9B). That is, this 1–2 m thick, 60 m long cross bed was moving up the slope of Mt. Sharp (Fig. 9B). Most importantly, foreset beds of this cross bed show abundant hummocky cross stratification (Fig. 9B). In addition, each foreset bed consists of one thick sandstone interval (30–50 cm) that is overlain by a thin (1–3 cm) mudstone interval (Fig. 9E).

About 30 m uphill (southward) along the same outcrop (Fig. 9A), the GHSb begins with a sandstone with trough cross beds with opposing dip angles which are bounded by large symmetrical ripples (Fig. 9C). This lithology is overlain with the sandstone layer with large south-dipping cross beds (Fig. 9C).

An additional 30 m uphill or southward (Fig. 9A), the GHSb begins with north-dipping layers of sandstone (Fig. 9C) which are truncated sharply and overlain first by near horizontal sandstone layers that clearly onlap them (Fig. 9C). This is followed by a thin interval of sandstones with cross beds with opposing dip angles (Fig. 9C). Finally, a sandstone with large south-dipping cross beds occurs at the top of the GHSb (Fig. 9C).

**Figure 10:**
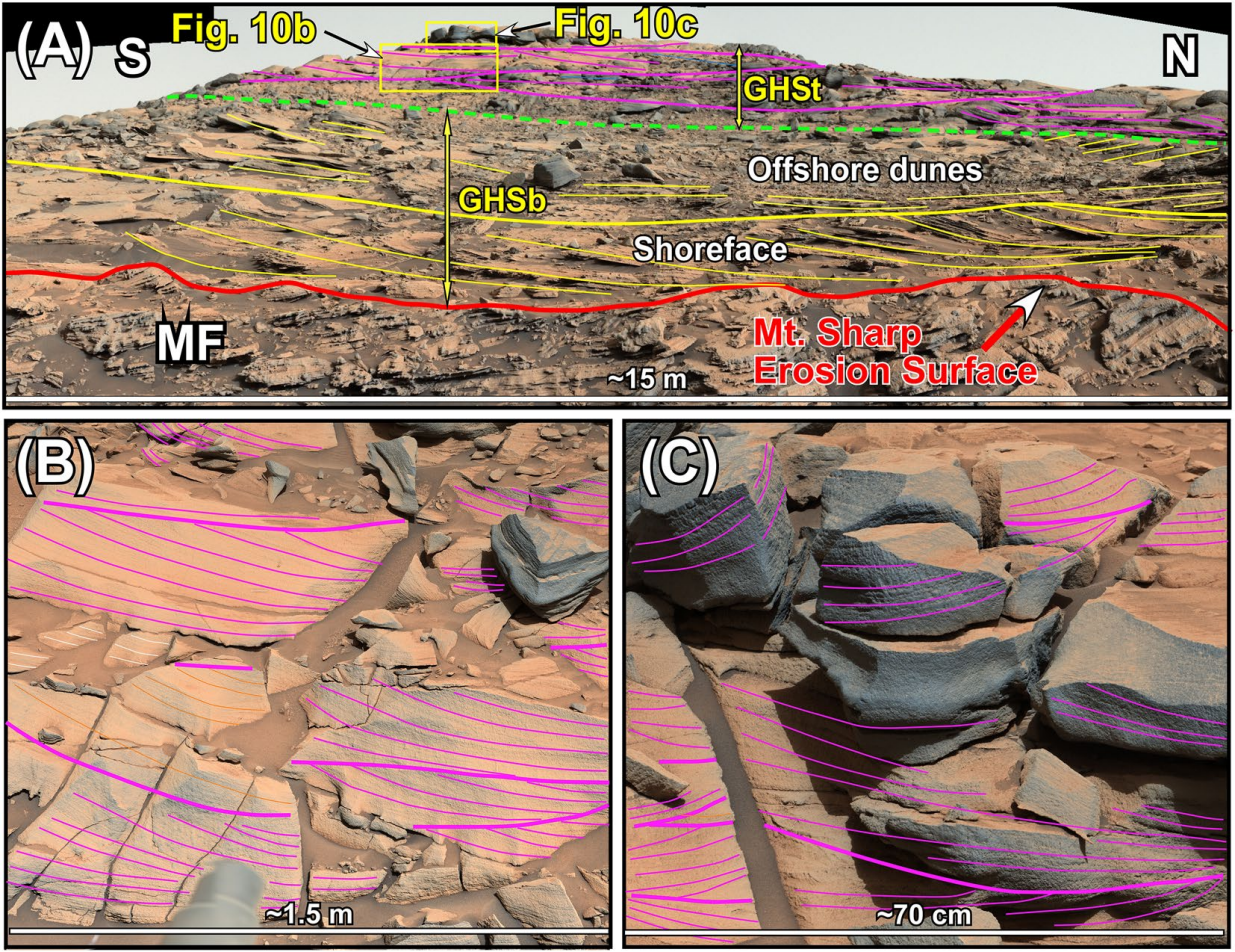
(**A**) Mastcam image mosaic shows internal features of one antidune ridge that covers the top of the Greenheugh patch. The antidune shows the basal and the top members, GHSb and GHSt, respectively, of the Greenheugh sandstone. The north-dipping layers at the base of the ridge are interpreted as beach deposits of the GHSb. They are overlain by south-dipping large cross beds of off-shore dunes. The foreshore layers were not preserved because they were eroded by the advancing offshore dunes. The antidune of the GHSt consists of lens-shaped packages of cross-bedded layers. The ridge is capped by lens-shaped siltstone. (**B**–**C**) Enlarged portions of Fig. 10A show internal bedforms of two layers within the antidune of the GHSt. Yellow and purple lines trace layering. Unprocessed images used to generate mosaics of this figure are publically available at the Planetary Data System web site at https://pds-imaging.jpl.nasa.gov/. The credit for Mastcam mosaic images of this figure goes to Malin Space Science Systems and NASA/JPL. Please see the Supplemental Document 1 for additional information on these images.

The GHSt directly overlies the GHSb with a sharp, north-sloping truncational contact (Fig. 9A–D). The GHSt shows one of the most distinctive sedimentological features in Gale crater: A field of symmetrical ridges that covers the entire surface of the Greenheugh patch (Fig. 8B). These ridges are about 100 m long, 1 m high, and have a spacing of about 15 m (Fig. 8B). They are so large that they were originally identified from the Martian orbit and were referred to as the Washboard^18^. Two ridges were imaged by the rover along the 70 m-long outcrop (Figs. 9B, 10). Internal sedimentary structures of both ridges are identical (Figs. 9B, 10). Each ridge is made up of amalgamation of lens-shaped packages of sandstone layers which dip mostly northward and occasionally southward (Figs. 9B, 10). This pattern of cross bedding is also visible in individual sandstone layers within each lens-shaped package (Fig. 10B).

## Discussion

Previous fluvial interpretation of the RTU^7^, and the aeolian suggestions for the deposition the SF^42^ and the GHS^70^ are not supported by our observations. Although not mentioned by name, outcrops of the RTU were previously interpreted as fluvial strata that were deposited by south-flowing waters that originated from the northern rim of Gale crater before the emergence of Mt. Sharp^7^. The following observations do not support this depositional environments for the RTU. (1) Fluvial deposits display some or all of the following sedimentological characteristics: abundant cross bedding, current ripples, channel morphologies, lag deposits, and desiccation cracks^72^. These features are not consistent with sedimentological and lithological characteristics of the RTU such as laminated siltstone to fine-grained sandstone, massive-bedded sandstone, matrix supported massive conglomerates (Figs. 3, 4, 6), ridge-shape bedforms (Fig. 6B), cross beds that dip both uphill and downhill (Fig. 6B), and the horizontal alignment of the flat-pebbles in conglomerates (Fig. 6D–F). (2) Fluvial deposits commonly fine upward^72^. This contrasts sharply with the coarsening upward grain size distribution of the RTU at the Cooperstown, the Dingo Gap, and the Kylie locations (Figs. 3A,B, 4). (3) The RTU was deposited on a surface that slopes northward (Fig. 2). This means that a south-flowing river, as proposed by previous authors^7^, is unlikely because the river was required to flow uphill.

The SF has been interpreted as a single aeolian erg that formed only along the foothills of Mt. Sharp and nowhere else in Gale crater during a cold and dry climatic episode of the Hesperian^42^. Our observations contradict this interpretation. In addition to the absence of interdune deposits^42^, virtually every characteristic of the SF in our study area at the Pahrump Hills locality contrasts sharply with those of unambiguous aeolian strata on Earth and in Gale crater. Here are a few examples: (1) The SF is composed of poorly sorted coarse-grained sandstone to pebbly sandstone (Fig. 5B); whereas, aeolian sediments consist overwhelmingly of well-sorted, fine to medium grain sand (Fig. 5C) on Earth and in Gale crater^73–76^. (2) The SF has two distinct members. The SFb displays large cross beds that moved up the slope of Mt. Sharp or southward (Fig. 7A,B) whereas sandstone layers of the SFt display bundles of cross beds that moved down the slope of Mt. Sharp or toward north (Fig. 7C,D). Flow directions derived from the SFb and the SFt contrasts sharply with the modern aeolian deposits in Gale crater that migrate exclusively towards southwest^75,76^. (3) Most importantly, the SFt in the Pahrump Hills area transitions into thin-bedded, horizontal strata of the DM (Fig. 7D). The implication of this discovery is enormous. It suggests that the SFt and the DM are time-equivalent: formed side-by-side at the same time. In addition, it indicates that deposition at the foothills of Mt. Sharp was linked to deposition on Aeolis Palus, and both are components of one depositional environment: a lacustrine system (see below).

The patch of the GHS (Figs. 1C, 8A) that occurs on the slope of Mt. Sharp was previously considered to be the extension of aeolian erg that formed the SF ^70^. The SF and the GHS share several stratigraphic similarities. Both overlie the MF with sharp erosional contacts (Figs. 7A, 9A–D). Both consist of a basal and a top member: the SFb and the SFt (Fig. 7) versus the GHSb and the GHSt (Fig. 9). Basal members in both rock units (SFb and GHSb) consist of large cross beds that migrated up the slope of Mt. Sharp or southward (Figs. 7A, 9B); their top members (SFt and GHSt) are composed of sandstone whose cross beds show flow downhill on the slope of Mt. Sharp or toward the north (Figs. 7C,D, 9B). These similarities suggest that the SF and the GHS are time equivalent (formed at the same time), as was also concluded by previous authors^70^. This conclusion implies that deposition on the Greenheugh patch, on the foothills of Mt. Sharp, and on Aeolis Palus are related. In addition, features that negate aeolian deposition of the SF and discussed in previous paragraphs also apply to the deposition of the GHS suggesting that neither the SF nor the GHS were deposited in an aeolian erg environment.

### Proposed interpretations

**Figure 11:**
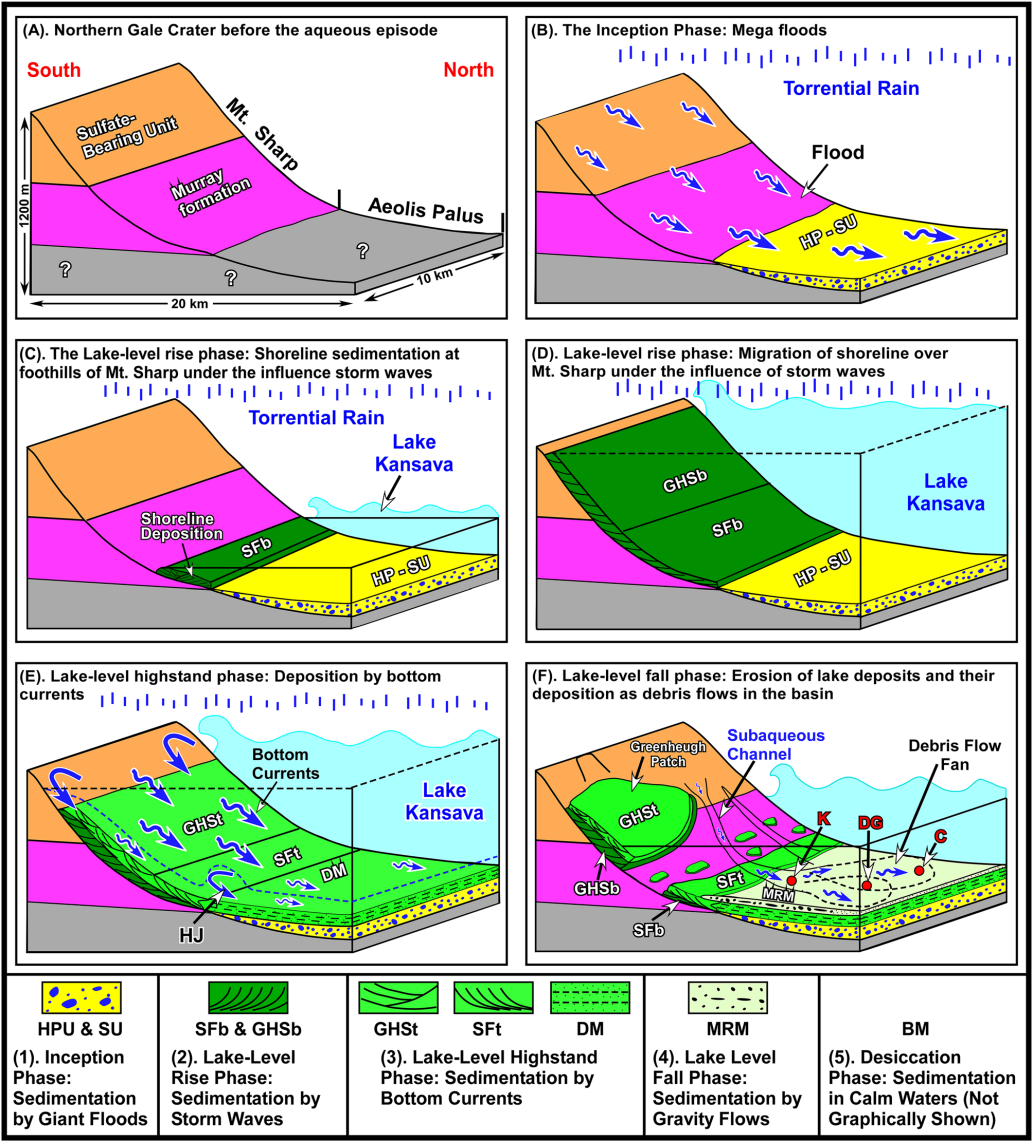
(**A**) Schematic diagram shows the northern part of Gale crater before the aqueous episode. (**B**) The inception phase sedimentation was associated with deposition of the Hummocky Plains Unit (HPU) and the Striated Unit (SU) on Aeolis Palus^8^. Floods formed by planet-wide torrential rain that flowed into Gale crater and also roared downhill from Mt. Sharp. (**C**) Lake Kansava formed in the lowest elevations of Gale crater and expanded so quickly that no sedimentation occurred until its shoreline reached the foothills of Mt. Sharp. The lake-level rise phase sedimentation began there and deposited the basal member of the Stimson formation (SFb) under the influence of storm waves. (**D**) The lake-level rise phase sedimentation continued as the shoreline migrated up the slope of Mt. Sharp and deposited the basal member of the Greenheugh sandstone (GHSb) under the influence powerful storm waves. (**E**) The lake-level highstand phase sedimentation began. Return flow from pounding of strong waves against Mt. Sharp formed strong bottom currents that flowed downhill and deposited top member of the Greenheugh sandstone (GHSt) as a 3 km-long field of large antidunes (the Washboard). Bottom currents were decelerated at the foothill of Mt. Sharp because of a major change in slope and the formation of a hydraulic jump (HJ). It resulted in the deposition of top member of the Stimson formation (SFt). Bottom currents continued downhill to form the Dillinger member (DM) on the crater floor. (**F**) Lake-level fall phase scoured transgressive and highstand strata and re-deposited them as sandstone and conglomerate in subaqueous channels and debris flows fan of the Mt. Remarkable member (MRM) on the basin floor.

Our observations indicate that the RTU, the SF, and the GHS are neither fluvial nor aeolian. They did not occur in two separate times, did not deposit in two different environments, and did not form under two opposite climate systems. We document that these three rock units were deposited in a 1200 m-deep paleolake under the influence of powerful storm waves (Fig. 11). Our conclusions are based on observations that the Curiosity rover made along its traverse on a surface that once was the bottom of this lake (Figs. 1C,D, 2). As such, the rover systematically examined strata that were deposited in the deepest waters of the paleolake on the northern part of the crater floor (Aeolis Palus) to layers that formed along its shoreline on Mt. Sharp (Figs. 1C, 2, 4). This provided a rare opportunity to document the evolution of one aqueous episode from its inception to its desiccation and to determine the type of warming event that caused it. Such a study provides a window into geologic, hydrologic, and climatic conditions of Mars when it was a warm and wet planet. Our study indicates that the aqueous episode we investigated had five phases of development and each phase was defined by specific sedimentological processes (Figs. 4, 11). They are discussed in a chronological order, from the oldest (A) to the youngest (E), and include the following:

A. The inception phase: sedimentation by giant floods
B. The lake-level rise phase: shoreline sedimentation by storm waves
C. The lake-level highstand phase: sedimentation by bottom currents
D. The lake-level fall phase: sedimentation by sediment gravity flows
E. The desiccation phase: sedimentation in calm waters of the lake

Please note that, each phase is a duration of time and a part of a continuum of one lake-level change. We also identified, strata that deposited in each phase of this one lake-level change.

### The inception phase: sedimentation by giant floods

This phase demonstrates how the aqueous episode began. Our previous study^8^ on detailed sedimentological characteristics and depositional environments of the Hummocky Plains Unit (HPU) and the Striated Unit (SU) provided the answer. The RTU, one of the lacustrine deposits (see below), overlies the HPU and the SU with sharp contacts over the entire area of its exposures on Aeolis Palus (Figs. 1D, 3, 4). Here is a summary of our investigation of the HPU and the SU^8^.

The HPU forms smooth surface hummocks. It is always overlain by thin beds of the DM of the RTU with a sharp contact (Figs. 3, 4). Examinations of its exposures at the Cooperstown, the Dingo Gap, the Kylie, and the Kimberley areas (Fig. 1B–D) revealed that the HPU is composed of a poorly sorted cobble to boulder conglomerate^7,8,25,29^. In addition, this rock unit displays linear, symmetrical ridges, which are about 10 m high and occur at a constant spacing of 150 m^8^. The HPU also shows cross beds that are 2 m to 6 m thick and up to 60 m long^8^.

The cobble to boulder grain size, the 60 m long and 2–6 m thick cross beds suggest that the HPU conglomerate was deposited by powerful currents capable of moving such large fragments^8^: giant floods. The 10 m-high symmetrical ridges of the HPU were interpreted as antidune: a type of bedforms that deposits only when the current is flowing very fast as determined by the Froud number (F) that is equal or greater than one^8,77^. The 150 m spacing of these antidunes suggests that flood waters that deposited the HPU were 24 m deep at the Kimberley location^8^. The orientation of the symmetrical ridges, the current direction derived from cross beds, and the deposition of the HPU on a north sloping surface suggest that the flow that deposited the HPU roared down the slope of Mt. Sharp^8^ (Figs. 4, 11A,B).

The SU outcrops consist of large numbers of individual patches that overlie hummocks of the HPU (Fig. 1D). These patches are concave upward and are made up of south-dipping layers that strike N60°E and have an estimated dip between 0° to 10° SE^7,8,25,29^. Patches are 100 m to 200 m along their strike and 50 m to 100 m along their dip^8^. Each patch consists of 5 to 10 layers and each layer is composed of pebble conglomerate at the base that grades to medium-grained sandstone at the top^8^. The SU patches always occur on the southern limb of the HPU ridges^8^. The consistent association between the occurrence of the HPU and the SU patches suggests that deposition of the two rock units is linked together^8^. The investigation concluded that the SU patches formed by the erosion of antidunes of the HPU as flood waters decelerated^8^.

The study of the HPU and the SU resulted in three very important conclusions that revealed how the inception of the aqueous episode we studied took place. First, the HPU and the SU were deposited by giant floods inside Gale crater^8^.

Second, flood waters that deposited these two rock units roared down the slope of Mt. Sharp^8^. Please noted that Mt. Sharp is a mound in the middle of Gale crater (Fig. 1A). It is not connected to the Martian mainland (Fig. 1A). Therefore, water that flowed down its slope could not have been supplied by rivers from the Martian highlands. This means that the water that roared down the slope of Mt. Sharp and deposited the HPU and the SU (Fig. 11B) must have been the result of the tremendous torrential rain fall over Gale crater. This conclusion also implies that floods that drained into Gale crater through inflow channels, were also due to torrential rain fall over the Martian mainland. In other words, the rainfall was planet wide^8^.

Third, the occurrence of the HPU and the SU directly beneath strata of lacustrine origin such as the RTU (see below) suggests that the inception of the aqueous episode we investigated began by the sudden and catastrophic arrival of floods into Gale crater. Flood waters entered the crater through numerous channels on the crater rim, the most important of which is Farah Vallis: a one-km wide, 700 m deep inflow channel (Fig. 1A). Gale crater is a closed basin: it does not have an outflow channel (Fig. 1A). Flood waters that entered the crater from the Martian highland and those that roared down Mt. Sharp accumulated in areas with the lowest elevation that was and is near the Yellowknife Bay area forming a lake there (Fig. 11C). This lake is informally named Lake Kansava^78^. Also, the inflow of rainfall-driven floods eventually formed a 1200 m deep lake in a 154 km wide crater. This suggests that the rain fall was not an ordinary, localized rain shower. It must have been wide spread and possibly occurred over the entire planet.

### The lake-level rise phase: shoreline sedimentation by storm waves

This phase reveals the evolution of lake-level rise of Lake Kansava. Flood deposits of the HPU and the SU are overlain by the thin-bedded siltstones of the DM of the RTU throughout their exposures (Figs. 3B,D,E, 4). Sedimentological characteristics of siltstone layers of the DM suggest that this rock unit was deposited in deep water (see below). That is, deep water siltstones of the DM directly overlie conglomerate of the HPU that were deposited by floods^8^ (Figs. 3B,D,E 4). The absence of shallow water lacustrine strata between the HPU and the DM on Aeolis Palus (Figs. 4, 11D) indicates lake-level rise was not slow and gradual. Otherwise, shallow water lacustrine strata would have been deposited and would have been preserved. The fact that no sedimentation took place on Aeolis Palus when lake-level was rising suggests that the lake expanded quickly and the lake-level rise was so rapid that no shallow water lacustrine sedimentation deposited on Aeolis Palus (Figs. 4, 11C). That is, a gap exists at the boundary and the contact between the DM and the HPU is disconformable. This is a common characteristic of sedimentation in oceans and lakes on Earth when the sea-level or the lake-level rise quickly^79–81^.

The lake-level rise without sedimentation continued until the lake’s shoreline reached the foothills of Mt. Sharp (Figs. 4, 11C). Lacustrine sedimentation began at that location along the shoreline of Lake Kansava for the first time (Figs. 4, 11C). This is clearly confirmed by sedimentological features of the SFb that outcrops in this area (Figs. 4, 7A,B, 11C). The preserved lithology of the SFb begins with a layer of sandstone with trough cross beds that shows opposing dip angles (Fig. 7A,B) indicating deposition by opposing flow directions. This type of cross bedding suggests sedimentation by waves in a shoreface environment, as documented extensively in such environments on Earth^82,83^. The shoreface sandstones of the SFb are overlain by layers of sandstone with large (0.5–1 m high) cross beds indicating that the sand was moving up the slope of Mt. Sharp or southward (Fig. 7A,B). The application of Walther’s Law^84^ suggests that sandstone layers with large (0.5–1 m) cross beds must have formed as large (meter high) offshore dunes that migrated (advanced) over the shoreface as the shoreline moved up the slope of Mt. Sharp during the lake-level rise (Fig. 11C).

Sedimentological features that characterize the SFb at Pahrump Hills (Figs. 4, 7) also occur in the GHSb at the Greenheugh patch (Figs. 1C, 4, 8A). As such, the stacking pattern of lithologies and sedimentary structures of the GHSb (Figs. 4, 9) are also indicative of deposition along a shoreline that was moving uphill over the slope of Mt. Sharp. This is known as a transgressive shoreline deposit, similar to those seen on Earth^82,83^. However, the preserved lithology of the GHSb is a much more complete record of a shoreline than that of the SFb (Fig. 4). The north-dipping thin beds at the base of the GHSb (Figs. 4, 9D) are the foreshore (the beach); the overlying sandstone containing cross beds with opposing dip angles and symmetrical ripples (Figs. 4, 9C) represent the shoreface; and the top sandstone with large cross beds (Figs. 4, 9B) are indicative of meter-high offshore dunes (Figs. 4, 9A). The similarity between the sedimentology and depositional environments of the SFb (Figs. 4, 7) and the GHSb (Figs. 4, 9) indicates that the shoreline that formed at the foothills of Mt. Sharp and deposited the SFb moved uphill over Mt. Sharp for 6 km as lake-level rose and deposited the GHSb at the location where the Greenheugh patch is currently located (Fig. 11C,D).

The near perfect preservation of the sedimentological record of the GHSb along the 70 m-long exposure allows the reconstruction of lake-level fluctuations in Lake Kansava (Fig. 9). The north-dipping surface on which the GHSb was deposited, the top of the MF, was the lake’s bottom (Fig. 9A). It can be considered a depositional timeline^79–81^. This allows us to determine time equivalent strata of shoreline deposits the GHSb. The lake-level change along the 70 m long outcrops occurred as follow (Figs. 4, 9). An initial lake-level rise resulted in the movement of the shoreline up the slope of Mt. Sharp (southward) to the point P1 (Fig. 9A). After that, the beach began to retreat (Fig. 9D) until it reached the point P2 (Fig. 9A). At that time, the foreshore was located at the point P3 and offshore at the point P4 at a water depth of about 2–3 m (Fig. 9A). This water depth is estimated based on the elevation difference between the beach facies and the offshore along the time line (Fig. 9A). Soon after, a subsequent major lake-level rise advanced large offshore dunes uphill (Fig. 9B). This resulted in the erosion and cannibalized the shoreface and foreshore strata as offshore dunes continued to moved up the slope of Mt. Sharp (Fig. 9B–D). That is, one large lake-level rise was punctuated with a small retreat, that is also typical of lake-level or sea-level changes on Earth^79–81^. Our observations indicate that the SFb and GHSb were deposited by storm waves (see below) which are considered to short duration events; and their deposition lasted during the lake-level rise.

A major issue in Martian geology has been whether waves occurred in oceans and lakes of the red planet^85^. Experimental and theoretical studies indicate that it could have^85,86^. And, waves on Mars were influenced primarily by the atmospheric pressure, the wind speed, the fetch, and the Martian gravity. These studies also conclude that waves form under any atmospheric pressure on the red planet^85,86^. However, wave height and wave speed are influenced by the low gravity of Mars which is about one-third that of Earth^85,86^.

The occurrence of waves allows us to estimate the water depth at which offshore dunes of the SFb and the GHSb formed via the relation between dune height (H) and water depth (h) ^87^:

1 $${\text{H }} = \, 0.{167}\,{\text{h}}$$

As such, deposition of meter-high dunes requires a water depth of about 5–6 m on Earth. However, the wave base on Mars would have been one-third the value of that on Earth^88^ indicating that meter-high offshore dunes of the SFb and the GHSb formed in water depth of about 2–3 m in Lake Kansava. This calculated water depth deposition of offshore dunes of the GHSb is similar to one we estimated by graphical procedures (the elevation difference between points P2 and P4 in Fig. 9A).

The most important question about shoreline deposits of the Lake Kansava is how did meter-high offshore dunes (Figs. 7A,B, 9B–D, 11C) moved 6 km uphill over the slope of Mt. Sharp during the lake-level rise? Four possibilities exist. The first is that low Martian gravity would have facilitated grain transport. Therefore, ordinary waves in Lake Kansava not only would have been able to form meter-high dunes in offshore environments but also were capable of moving them up the slope of Mt. Sharp. We consider this possibility unlikely. This is why. The average daily wind speed in the present-day Gale crater is about 5 m/s or 18 km/h^65,66^. This wind is not strong enough to move micron size dust particles that cover everything in Gale crater. For this reason targets examined by the Curiosity rover are often brushed by the Dust Removal Tool (DRT) for viewing. This fact indicates that ordinary wind speed in Gale crater 4 billion years ago would not have been able to produce strong enough waves to move clay-size particles even under the low gravity of the red planet.

Second, formation and movement of large dunes took place regularly under the influence of tidal waves in tidally influenced environments on Earth^87^. We consider this possibility unlikely for Lake Kansava, because it was too small of a water body for tidal waves to form even if a large Martian moon existed.

Third, meter-high dunes formed and moved up the slope of Mt. Sharp by large waves that were produced by the impact of asteroids into Lake Kansava, similar to the proposed formation of such waves in a Martian ocean^89^. This interpretation is also unlikely, because foreset layers of large cross beds observed in the SFb and the GHSb are highly rhythmic (Fig. 9E) suggesting that the mechanism that produced them occurred regularly. In addition, meter-high dunes formed at the bottom of Mt. Sharp and moved over 6  km uphill. This could not have happened by a single or even a few asteroid impacts.

Fourth, meter-high offshore dunes of the SFb and the GHSb formed and moved up the slope of Mt. Sharp by strong waves which were produced by powerful storms that passed through the area regularly. We consider this possibly the most likely. Not only because storms commonly deposit large dunes on Earth^90,91^, but also because meter-high offshore dunes of Lake Kansava display features that support their deposition by storm waves. These include the following. (1) The sandstone—mudstone alternation in foreset beds of these large cross beds recorded the presence of such storms (Fig. 9E). The thick (5–30 cm) sandstone interval (Fig. 9E) was deposited during the active passing of storm waves that were strong enough to move the dune up the slope of Mt. Sharp (Fig. 9C). The thin (1–3 cm) mudstone to siltstone layers that overlie the sandstone beds (Fig. 9E) deposited from suspension in calm waters after the passing of storm waves. (2) Hummocky cross stratification are abundant in the foreset beds of these cross beds (Fig. 9B). It is well known that hummocky cross stratification is an indicator of deposition by storm waves^91,92^.

### The lake-level highstand phase: sedimentation by bottom currents

This phase shows how high the shoreline advanced on Mt. Sharp, how deep the lake became, and what sedimentation processes occurred at that time. The highest elevation where outcrops of shoreline deposits of the GHSb are preserved is located at the southern edge of the Greenheugh patch at an elevation of about − 3900 m: the − 3900 m shoreline (Fig. 1B). The deepest area of Gale crater then and now is located at the elevation of − 4500 m at the YKB area (Figs. 1D, 2). This suggests that Lake Kansava was at least 600 m deep when the shoreline was at − 3900 m (Fig. 1B). However, geomorphic evidence suggests that lake-level rise did not stop at − 3900 m elevation. It continued to rise although the shoreline deposits were subsequently eroded. This conclusion is reached because of two geomorphic evidence. First, the sudden termination of channels along the − 3300 m elevation around the perimeter of Mt. Sharp (Fig. 1A). Second, similar features that occur around the crater rim^16^. These observations indicate that the lake level advanced to − 3300 m: the − 3300 m shoreline (Fig. 1A). This elevation is referred to as the peak expansion of the lake or the lake-level highstand. Lake Kansava was about 1200 m deep at the Yellowknife Bay area when its shoreline was at − 3300 m (Fig. 1A).

Storm waves continued to pound against Mt. Sharp eroding its sediments as they did during the lake-level rise. Eroded sediments that were produced during the lake-level rise would have been trapped at the shoreline to form the beach by the rising lake-level. At the lake-level highstand, however, eroded sediments could not be stopped at the shoreline because the preventing force of lake-level rise no longer existed. Therefore, sediments would have moved downhill toward deep waters by the return flow that was produced by the pounding of storm waves against Mt. Sharp, similar to the landing of hurricanes on Earth^93,94^. Bottom currents and gravity flows are two major mechanisms of sediment transport to deep waters in lacustrine and marine systems on the terrestrial planet^93^. The absence of Bouma sequence (a signature feature of turbidites^95^) in any of the rock units that were deposited during this phase of the lake evolution (see below) suggests that the transport of sediments to deep waters of Lake Kansava took place by bottom currents.

Two out of the three rock units that were deposited during the highstand phase of sedimentation are the GHSt and the SFt (Figs. 4, 11E). This conclusion is reached based on the stratigraphic analysis of these two rock units and the flow direction of water that deposited them as was determined from their sedimentary structures. The GHSt and the SFt overlie strata that were deposited during the lake-level rise with sharp erosional contacts, namely the GHSb and the SFb, respectively (Figs. 4, 7A, 9B, 11E). That is, the contact between the GHSt and GHSb, and that of the SFt and SFb are disconformable. Cross beds within both the GHSt and the SFt show flow direction down the slope of Mt. Sharp toward north (Figs. 4, 7A, 9B). That means that sediments were moving downhill over the slope of Mt. Sharp for the first time. These two observations indicate that the GHSt and the SFt were deposited when lake-level rise had ended and lake-level highstand had begun (Fig. 11E).

The third rock unit that was deposited during the lake-level highstand phase is the DM of the RTU (Figs. 4, 11E). The DM does not overlie any transgressive strata because the fast rising of lake-level over low-slopes of the Aeolis Palus did not leave behind sediments there. The DM consists of layers of siltstone to fine-grained sandstone (Fig. 5A) that are centimeter-thick (Fig. 6A,B) and show a uniform lithology over 3 km of continuous outcrop examined by the rover (Fig. 3A,B,D,E). These sedimentological characteristics are typical of deposition in the deep waters of a lake (Fig. 1A) as are commonly observed in deep-water fluvial-lacustrine strata on Earth^80,81^. In addition, sedimentary structures of the DM, namely plane laminations, 10–30 cm high symmetrical ridges, and cross beds that dip downhill and uphill (Fig. 6A,B) indicate that the DM deposited by moving currents. In fact, symmetrical ridges and cross beds of the DM are identical to the internal structure of the antidues as documented extensively by flume studies^96,97^. This suggests that the deposition of siltstone and fine-grained sandstone of the DM in deep waters of Lake Kansava occurred by fast moving flows at the bottom of the lake: bottom currents. As such, the same bottom currents that deposited the GHSt at the Greenheugh patch and the SFt at the foothills of Mt. Sharp, continued downhill to deposit the DM in the deepest waters of Lake Kansava on Aeolis Palus (Figs. 1C, 2, 4, 11E).

A close examination indicate that sedimentary structures of the three rock units that deposited during the lake-level highstand (the GHSt, the SFt, and the DM) slightly differ (Figs. 4, 11E). This can be attributed to the changing nature of bottom currents along its flow path. For example, the GHSt displays a 3 km-long field of symmetrical ridges over the Greenheugh patch (Fig. 8B). These ridges were deposited on a north-sloping surface that truncates the underlying strata of the GHSb (Fig. 9A–D). Internally, these ridges are composed of amalgamation of lens-shaped packages of strata (Figs. 9B, 10). Each lens consists of layers that dip primarily downhill (toward the north) and secondarily uphill or toward the south (Figs. 9B, 10). Note that sedimentary structures of the GHSt (Fig. 9C, 10) are just a taller and a larger version the ones in the DM (Fig. 6B) because of the difference in grain size between these two rock units: siltstone in the DM (5A) vs medium grain size in GHSt (Fig. 5D). Therefore, the same analogy that we used to interpret sedimentary structures of the DM also applies here. As such, symmetrical morphology and internal sedimentary structures are classical features of formation and destruction of antidunes as documented extensively by flume studies^77,96,97^. The absence of mud cracks and the lack of any feature indicative of subaerial exposures suggest that antidunes of the GHSt were deposited in a subaqueous environment (Fig. 11E), as commonly seen on Earth^97,98^. The lack of Bouma sequence (signature feature of gravity flows^95^) in layers that make-up the antidunes (Figs. 9D, 10) suggests that they were deposited by powerful (supercritical) bottom currents (Fig. 11E). This is also similar to the formation of these structures on Earth^98^.

The GHSt and the SFt display similar stratigraphic positions although they are not physically connected (Fig. 4). Both overlie strata that were deposited during the lake-level rise (Figs. 4, 11C): the GHSb and the SFb, respectively. This suggests that deposition of the GHSt is related to that of the SFt. As such, after depositing the GHSt in up-flow regions where the Greenheugh patch is located, bottom currents continued downhill and deposited the SFt at the foothills of Mt. Sharp (Figs. 7C,D, 11E). However, cross bedded sandstones of the SFt are indicative of deposition by subcritical flows: currents with the Froud number of less than one^77,96,97^. The transition from a downhill moving supercritical flow to a subcritical flow occurred because of the rapid decrease in slope angle at the foothills of Mt. Sharp (Figs. 2, 11E). This transition produced a hydraulic jump (Fig. 11E) as commonly occurs under such conditions on Earth^97^. Therefore, the supper critical bottom currents decelerated to subcritical flow that deposited cross-bedded sandstones of the SFt (Fig. 7C,D).

The SFt transitions into the DM at the base of Mt. Sharp (Fig. 7D). This suggests that the SFt and the DM were deposited in deep water by the same bottom currents. As such, bottom currents must have continued to travel downhill and deposited the DM on Aeolis Palus. This conclusion is supported by lithological characteristics of the DM and its sedimentary structures as discussed above. To summarize, our detailed sedimentological and stratigraphic study suggest that the layers of the GHSt, the SFt, and the DM were deposited by bottom currents which are short duration events. Deposition all three rock units began when the lake-level reached its highstand and terminated with the lake-level highstand ended.

### The lake-level fall phase: sedimentation by gravity flows

This phase incorporates sedimentary processes that occurred during the lake-level fall. Eventually, lake began its retreat most likely because of the slowdown in runoff (Fig. 11F). The MRM of the RTU is the only rock unit that deposited during this phase of sedimentation (Figs. 4, 11E). This conclusion is reached because of the stratigraphic position of the MRM and its distribution in the depocenter: Lake Kansava (Fig. 4). The MRM deposited over the highsand strata of the DM (Figs. 4, 6A,C). The contact between them (Fig. 3A,B,D,E) shows soft sediment deformation that resulted from scouring of some layers of the DM during the deposition of the MRM (Fig. 6C). The regional nature of the contact between the DM and the MRM is disconfomable (Fig. 3B–E), but scouring generated slightly angular boundary at the Cooperstown location (Fig. 3A). This also indicates that the MRM deposited in a subaqueous environment when the DM strata were still soft (see Fig. 6C). However, in contrast to the DM that transitions into the SFt at the foothills of Mt. Sharp (Figs. 4, 7D), the MRM is present only on the crater floor (Fig. 4). This suggests that deposition of the MRM took place during a major basin-ward shift in sedimentation (Figs. 4, 11F). This pattern of deposition is indicative of a fall in lake-level or sea-level on Earth ^99^. The same conclusion applies to Mars and suggests that the MRM must have been deposited during the lake-level fall (Figs. 4, 11F).

Sedimentological characteristics of the MRM at the Cooperstown, the Dingo Gap, and the Kylie locations (Fig. 1C,D) consist of massive bedding, unsorted texture, a coarsening upward lithology, matrix-supported conglomerate, and orientation of plane pebbles parallel to the bedding (Figs. 2, 3A–D, 6D–F). These are classical features of sedimentation by debris flows: a type of sediment gravity flows that deposits under subaerial or subaqueous conditions^100,101^. This indicates that the pounding of storm waves must have continued during lake-level fall and partially eroded transgressive and highstand strata which resulted in their patchy distribution on Mt. Sharp (Figs. 1C, 11F). Eroded sediments were then transported downhill (northward), this time by gravity flows, and not by bottom currents. The eroded sediments were delivered to deep water thorough channels that extend from Mt. Sharp to the crater floor and were re-deposited in subaqueous channels and in debris flow fans on Aeolis Palus (Fig. 11F). Many channels occur on the slopes of Mt. Sharp (Fig. 1A,B) some of which can be linked to channels on the crater floor (Fig. 1B). One such example is shown in Fig. 1B. Here, a 1 km-wide channel that originates on Mt. Sharp can be traced to a channel on the crater floor (Fig. 1B). The Kimberley outcrop is located in this channel (Fig. 1B). Therefore, the fining upward grain size distribution of the MRM at the Kimberley outcrop (Fig. 4) and its linear morphological occurrence at this location (Fig. 3E) is interpreted as a subaqueous channel fill, identical to formation of such deposits on Earth^101^. However, the subaqueous channel deposit at the Kimberley area (Fig. 4) is now preserved as an inverted channel deposit (Fig. 3D), similar to those that occur on Earth and on Mars^102^. To summarize, our analysis of sedimentology and the stratigaphy suggest that the MRM was deposited by numerous individual subaqueous debris flows, a type of sediment gravity flow deposits, which are short duration events. The deposition occurred during the lake-level fall.

### The desiccation phase: sedimentation in calm waters of the lake

The desiccation phase represents a duration of time when Lake Kansava became a standing body of water and was experiencing desiccation. The one-meter thick BM of the RTU was deposited during this phase of lake evolution (Fig. 6F). This rock unit begins with cross bedded sandstone or siltstone at its base that grades upward to siltstone and sandstone with plane laminations (Fig. 6F). The BM overlies the conglomerate lithology of the MRM with a very sharp contact (Fig. 6F). Such sharp lithogical changes are usually an indication of missing geological record. Therefore, the contact between the BM and the RM is considered to be disconformable.

The sedimentological characteristics of the DM and its stratigraphic position above the MRM suggest that it was deposited in a subaqueous environment (Fig. 11F). Part of its deposition, particularly the cross bedded interval at its base, was most likely deposited by the suspended cloud of the flow that deposited the MRM, as commonly occurs in on Earth^101^ (Figs. 4, 11F). Its top strata with their plane laminations were deposited in the calm waters of lake. Deposition of the BM continued until Lake Kansava dried up. Cross bedded sandstone indicate that sediment delivery to the lake that was being desiccated continued, most likely by occasional torrential rains that produced flows from Mt. Sharp. The laminated nature of the DM indicates that its deposition occurred by short duration events that lasted during the desiccation phase of the lake.

Unfortunately, the BM was not accessible to the rover for close examinations. Therefore, we could not observe strata at the top of this rock unit to interpret the details of the desiccation process. However, we speculate how the desiccation could have occurred. It appears that storms ended after the deposition of the debris flow deposits of the MRM and waters of the lake became calm. Lake’s waters continued to evaporate and/or sublimate until the lake dried up. That is, the aqueous episode that began with the sudden and catastrophic pouring of giant floods into Gale crater ended calmly in the quiet waters of Lake Kansava. Desiccation did not leave behind any evaporites, suggesting the Lake Kansava was a freshwater lake.

## Lake Kansava and global warming on Mars

Three questions remain: when did Lake Kansava form, how long it lasted, and what triggered the warming mechanism that established this aqueous episode. The first question is linked to the age of Gale crater. As indicated in the introduction section, formation of Gale crater, its filling with sediments, and the subsequent erosion of its margins occurred during the Middle to Late Noachian time. This amounts to a duration of 350 million years^103^ of geological activity in Gale crater. Lake Kansava formed after the emergence of Mt. Sharp when Gale crater had acquired its modern morphology. Therefore, the most likely possibility is that the lake existed during the Late Noachian time and possibly towards the end of it.

How long Lake Kansava lasted is more difficult to determine than the age of the lake. Defining the duration of a geological process on Mars is difficult without reliable radiometric age dating of the beginning and the end of the event. However, a common method of speculation is a comparison with Earth, although terrestrial environments are not ideal analogs^104^. We have chosen East African lakes for comparison, where extreme rates of lake-level changes occur. Lakes in this area rise and fall from 10 to 150 m per 1000 years^105^. Lake Kansava experienced 1200 m of lake-level rise followed by 1200 m of fall. Assuming that rates of rise and fall were similar, this accounts to a duration of 16 Ky to 240 Ky. Late Noachian lasted for 140 million years^103^. As such, Lake Kansava existed for a fraction of one percent of the duration of Late Noachian. Even if our estimation has an error factor of ± 10 times, that would not significantly change this conclusion. This speculation highlights one point: Our investigation captured an event that was short-lived. This is consistent with the geological history of lakes on Earth^81^. This is because lakes are not small oceans^81^. The rate of change in lakes is very rapid and varies widely^81^. Lake-level change of 200 m in 10,000 years is common on Earth^81^.

The third question is the warming mechanism that facilitated the formation of Lake Kansava. This is related to a decades-old question: How to warm Mars. Proposed hypotheses include geothermal heating of the Martian crust^106^, CO_2_ clouds^107^, asteroid impacts^108^, high water salinity^2^, volcanism^109^, orbital changes^110,111^, and high methane concentration^112–114^.

None of these solutions are universally accepted. However, each may have been a contributing factor in climate change on Mars at one time. A major advantage of our investigation is that we examined a near complete sedimentological record (Fig. 4) of the aqueous episode that led to the establishment of Lake Kansava (Fig. 11). Our detailed study of these strata will provide clues to processes that took place during the formation and evolution of the lake. Such process-oriented observations will permit us to eliminate some proposed warming mechanisms and consider others that are viable options. For example, Lake Kansava was a freshwater lake. This finding eliminates the role of salinity^2^ as a controlling factor on aqueous episodes in this particular case. In addition, the absence of dropstones, a key indicator of glacial lakes^115^, suggests that Lake Kansava was not ice-covered. Plus, we did not see any ice-rafted fragments suggesting that rivers that fed the lake did not carry large blocks of ice. These observations suggest rivers that fed the lake did not originate from melting glaciers. That is, the Martian crust was not frozen or glaciers did not exist at the time Lake Kansava formed. These conclusions eliminate geothermal heating of a frozen Martian crust^106^ and the CO_2_ clouds hypothesis^107^.

Furthermore, lacustrine sediments of the Lake Kansava overlie cobble to boulder conglomerate of the HPU with a sharp contact (Fig. 3). The HPU conglomerate was deposited by giant flood waters that roared down the slope of Mt. Sharp^8^. Mt. Sharp is an isolated mound in the middle of Gale crater. It is not connected to the Martian mainland. The water that roared down its slope must have been produced by massive torrential rainfall inside Gale crater. This conclusion also implies that flood water that entered Gale crater from the Martian mainland must have been produced by torrential rainfall. As such, the warming mechanism that formed Lake Kansava must have produced enormous amounts of water vapor into the atmosphere for a short period of time. That water vapor poured down as rain upon cooling. This evidence eliminates any proposal that cannot produce such a rapid production of water vapor. This includes the suggestion of a single gas like CH_4_ as the cause of warming^112–114^. Like any other greenhouse gas, methane could contribute to warming of Mars but it could not warm Mars by itself.

The orbital change mechanism that is proposed for warming Mars^110^ is also a well-known cause of climate change on Earth^116^. As a result, it is an extensively studied mechanism and its consequences are well known^116^. Variations of orbital parameters (precession, obliquity, and eccentricity) will cause changes in radiation budget of Earth’s polar regions^116^ which will result in alternating cold and warm episodes at regular periodicities that produce alternating glacial and interglacial periods, respectively. Orbital forcing climate changes are highly rhythmic and are associated with repeated accumulation and melting of glacial ice^116^. As we demonstrated (Fig. 11), Lake Kansava experienced one lake-level rise followed by one lake-level fall (Fig. 11). Such a lake-level change corresponds to a onetime warming event followed by a onetime cooling event. In addition, water that formed Lake Kansava was produced by massive torrential rainfall, not by melting of glacial ice. Therefore, changes in orbital parameters could not have triggered the warming event that formed Lake Kansava.

Similarly, the proposed volcanism for warming Mars^109^ is also a commonly considered mechanism for global climate change on Earth. It is regularly applied to explain the cause of major biological crises of the Phanerozoic Earth. The Permian—Triassic mass extinction is just one of many examples^117^. The primary basis for the hypothesis is that volcanism causes global climate change due primarily to the emission of CO_2_ and secondarily to other gases such as SO_2_^118–121^. The problem is that volcanically emitted CO_2_ is not large enough to cause major global change^122^. In fact, CO_2_ is not produced at once, rather it is generated by many eruptions each 100–1000s of years apart^122^. Additionally, volcanic rocks are a large sink for atmospheric CO_2_ through chemical weathering^122,123^. Therefore, the effect volcanism on global climate change on Earth is a questionable idea. The same could be true about Mars.

We will show that the mechanism that can satisfy most of the processes we inferred from our sedimentological observations is a warming event that was triggered by an asteroid impact. The original proposed processes of such a mechanism^108,124^ have been confirmed and modified by advanced 3D models^125–127^. Asteroid impacts produce tremendous amount of heat that vaporizes surfaces waters, shallow groundwater, glaciers, release CO_2_ from solid reservoirs, produce large amounts CH_4_ from gas hydrate sources, and generate large amounts of SO_2_ from disintegration of sulfates^108,124,125,127,128^. These gases are injected into the atmosphere practically at once. As the atmosphere cools, precipitation begins at rates of 1 to 2 m/year that is equivalent to the rate of rainfall in tropical regions of Earth and can last up to two decades^125–127^.

Our sedimentological observations confirm predicted processes associated with a warming event that is triggered by an asteroid impact. For example, deposition of cobble and boulder conglomerates of the HPU prior to the establishment of Lake Kansava suggests that the aqueous episode was initiated by the sudden and catastrophic pouring of rainfall in Gale crater (Fig. 11A, B) and by implication over the entire Mars. In other words, the warming event was global. The asteroid impact is the only mechanism that can produce global warming of Mars^125–127^. The warming event did not stop until Lake Kansava became 1200 m deep (Fig. 11D). The lake-level began to fall afterwards (Fig. 11E) and continued until the lake desiccated. That is, Lake Kansava experienced one lake-level rise followed by one lake-level fall. This corresponded to one episode heating followed by one period of cooling, respectively. This pattern of heating and cooling is a characteristic of an asteroid impact (Fig. 11). Overall, processes we inferred from our sedimentological studies are consistent with events that are predicated for an asteroid impact^108,124–127^.

One potential shortcoming of the proposed asteroid impact hypothesis is the duration of the warming it produces ^108,124–127^. Although it depends on the size of the impact, such a warming mechanism is relatively short lived, lasting several years to decades^108,124–127^. Our speculation indicates that Lake Kansava existed for at least 10,000 years. We suspect other process could have extended the duration of the impact-induced warming event. For example, as precipitation falls on the planet surface that was covered by silicate melt, the water will vaporize again and returns to the atmosphere forming water clouds. This will keep the atmosphere warm for a longer period because water vapor is a greenhouse gas^125–127^. In addition, CO_2_, CH_4_, and possibly H_2_ that were released by the heat of the impact are considered an excellent greenhouse gas combination^128,129^. Their effect could have been combined with the effect of the impact and kept Mars warm much longer. Furthermore, it is plausible that the Martian atmosphere and surface become thermally coupled after the asteroid impact. Under this condition, altitude would have a major control on temperature^130^. In this case, low elevation areas of the Northern Lowlands would become the hottest places on Mar, and high elevation regions would remain cold year around^130^. A circulation pattern would have been established in which air and moisture would rise from the hot, Northern Lowlands area and would blow toward the cold highlands. This circulation pattern and the delivery of heat and moisture could have slowed down the cooling process. In addition, it supplied constant wind and moisture to Gale crater at a regular basis; and that could be the reason for the rhythmic layering observed in strata of Lake Kansava (Figs. 6, 7, 9).

The formation of Lake Kansava may not have been the only aqueous episode of the Middle to Late Noachian. Remote sensing investigations have discovered intense fluvial processes on different locations of Mars during this time interval^11,131,132^. It is possible that each of these aqueous episodes was caused by a separate warming event. However, it cannot be ruled out that they occurred at the same time as the one in Gale crater. Only reliable age dates can resolve this.

## Perspectives and significance

Our results should be viewed with the overall perspective of sedimentation in Gale crater. The aqueous episode we investigated constitutes about 30 m of over 5 km of sedimentary rocks that deposited in Gale crater^4,18^. In addition, our speculations indicate that Lake Kansava lasted for about a fraction of one percent of the duration of the Late Noachian. However, neither the thickness nor the duration of the event can be considered a factor in its significance. It is not clear how many and how often such short duration events took place during Early Mars. Was it just a onetime event, a perturbation, or occurred regularly?

Most importantly, our study provided a complete sedimentary record of one aqueous episode and discussed its five phases of development. Sedimentological interpretation of lacustrine strata provided a method to test the applicability of proposed mechanisms of warming Mars. So far, all proposed models of warming Mars are theoretical possibilities. We provided a method of testing these models by a process-oriented sedimentological interpretation of strata of the aqueous episode they leave behind. The application of such an approach showed the aqueous episode we investigated was triggered by asteroid impact. Similar approach could document warming by other mechanisms.

Lastly, our results emphasize that only a detailed in situ examination of strata in Gale crater or elsewhere on Mars can decipher the true climate Early Mars. Of course, such in situ investigation should be combined with theoretical and numerical modeling^2,133^ for best results.

## Summary and conclusions

Our investigation documented the history of one aqueous episode through detailed study of sedimentology of strata that deposited in a 1200 m-deep lake that formed in Gale crater after the emergence of Mt. Sharp nearly 4 billion years ago (Figs. 1A, 11A). The deep-water lacustrine siltstones overlie flood-deposited conglomerates (Figs. 2, 3, 4) with a sharp contact. This indicates that the aqueous episode began suddenly and catastrophically. Giant floods inundated Gale crater (Fig. 11B) and flood waters accumulated in areas with low elevations forming Lake Kansava (Fig. 11C). The lake expanded so quickly that no sedimentation took place until lake-level reached the foothills of Mt. Sharp where lacustrine sedimentation began along the lake’s shoreline (Figs. 2, 4, 11C). Deposition of meter-high dunes in offshore environments of the lake and their migration up the slope of Mt. Sharp during the lake-level rise suggest that sedimentation occurred under the influence of  powerful storm waves (Figs. 2, 4, 11C,D).

At lake-level highstand, the return flow from the pounding of storm waves against Mt. Sharp generated strong bottom currents that transported sediments downhill (Figs. 4, 11E) and deposited one of the most prominent sedimentological features in Gale crater: a 3 km-long field of perfectly preserved large subaqueous antidunes on Mt. Sharp (the Washboard). Bottom currents continued to travel downhill and deposited sediments at the foothills of Mt. Sharp and on the crater floor (Figs. 4, 11E).

Lake-level fall scoured some of the transgressive and highstant strata which resulted in their patchy distribution on Mt. Sharp (Figs. 1C, 11F). Eroded sediments were transported downhill by gravity flows and were re-deposited as sandstone and conglomerate in subaqueous feeder channels and in debris flow fans on the basin floor (Figs. 4, 11F). The lake desiccated afterwards. No aqueous sediments overlie strata of Lake Kansava. This suggests that the establishment of Lake Kansava was the last time liquid water flowed into Gale crater. The crater has been dry for about 3.6 billion years.

Sedimentological characteristics of the aqueous episode we investigated suggest that it was the result of a global warming event on Mars that was triggered by the heat generated by an asteroid impact. Strata belonging to the aqueous episode (the HPU, the SU, the RTU, the SF, and the GHS) overlie a major surface of erosion on the northern flank of Mt. Sharp (Fig. 2). This period of erosion of undefined duration is attributed to an aeolian process^134^. Torrential rain occurred planet wide, giant floods flowed on land, powerful storms took place in the atmosphere, and strong waves formed in lakes. Due to the absence of age dates, the exact duration of the warming event that resulted in the establishment of Lake Kansava cannot be determined with certainty. Our speculation indicates that it could have lasted for a period ranging from 16 to 240 Ky. This is a fraction of one percent of the 350 million year-duration of Middle to Late Noachian during which Gale crater was geologically active.

## Methods

The study was conducted by analysis of images taken by Mastcam and by Mars Hand Lens Imager (MAHLI) cameras. Mastcam cameras are multispectral imaging systems, which consist of two digital cameras mounted on the rover’s mast (1.97 m above the ground). The left camera (M-34) has a focal length of 34 mm and the right camera (M-100) with a focal length of 100 mm, yielding pixel scales of 0.22 and 0.074 mrad/pixel, respectively. Mastcams are capable of full color panoramic and stereoscopic measurements^135^. Mastcam images were used to delineate sedimentary structures, sedimentary facies, stratal bounding surfaces, sedimentary architecture, and determine dip directions of bedding. MAHLI is a 2-megapixel color camera with a focusable macro lens that is mounted on Curiosity’s robotic arm to investigate rocks and minerals in Gale crater^136^. It acquires in-focus images at working distances ranging from 2.1 cm to infinity. It is capable of resolving fine sand grains^136^. MAHLI and Mastcam images were used for sedimentological and textural characterization of targets.

### Supplementary Information

Supplementary Information.

## Data Availability

Data and images used in this investigation are publically available at the Planetary Data System web site at https://pds-imaging.jpl.nasa.gov/ and can also be requested from the corresponding author.
