# Supplementary material for: Lacustrine sedimentation by powerful storm waves in Gale crater and its implications for a warming episode on Mars
Source: Sci Rep. 2023 Oct 31;13:18715. doi: 10.1038/s41598-023-45068-5 (PMC10618461; doi:10.1038/s41598-023-45068-5)
Supplement: Supplementary file 1 — Supplementary Information. [file 41598_2023_45068_MOESM1_ESM.docx]

| **Figure** | **Target Name** | **Sol** | **Location** | **Camera** | **Image Number** |
| --- | --- | --- | --- | --- | --- |
| 3A | Cooperstown | 439 | Cooperstown | Mastcam | MR Mcam01789 |
| 3B | 360 Mosaic | 541 | Dingo Gap | Mastcam | MR Mcam02134 |
| 3C | Dingo Gap Ridge | 530 | Dingo Gap South Wall | Mastcam | MR Mcam02092 |
| 3D | Kylie | 551 | Kylie | Mastcam | MR Mcam02233 |
| 3E | Mt. Remarkable | 595 | Kimberley | Mastcam | MR Mcam02509 |
| 5A | Windjana | 612 | Kimberley | MAHLI | 0612MH0001930000203348R00 |
| 5B | Ivanhoe | 1092 | Marias Pass | MAHLI | 1092MH0001700000401026R00 |
| 5C | Gobabeb | 1228 | Namib Dune | MAHLI | 1228MH0001700000403450R00 |
| 5D | Assyant Window | 2703 | Greenheugh Patch | MAHLI | 2702MH0002270001002262R00 |
| 6A | Enlarged area in 3A | 439 | Cooperstown | Mastcam | MR Mcam01789 |
| 6B | Middle Unit | 601 | Kimberly | Mastcam | MR Mcam02537 |
| 6C | Enlarged area in 3A | 439 | Cooperstown | Mastcam | MR Mcam01789 |
| 6D | Workspace | 441 | Cooperstown | Mastcam | MR Mcam01813 |
| 6E, 6F | Enlarged area in 3C | 529 | Dingo Gap | Mastcam | MR Mcam02092 |
| 7A | West Wall Cliff | 753 | Pahrump Hills | Mastcam | MR Mcam03234 |
| 7B | Upheaval Dome | 751 | Pahrump Hills | Mastcam | MR Mcam03228 |
| 7C | Upheaval Dome | 751 | Pahrump Hills | Mastcam | MR Mcam03228 |
| 7D | Amargosa NW Wall | 748 | Pahrump Hills | Mastcam | MR Mcam03223 |
| 9A Left | Drive Direction | 2692 | Greenheugh Patch | Mastcam | ML Mcam14085 |
| 9A Right | Moray Firth | 2692 | Greenheugh Patch | Mastcam | MR Mcam13955 |
| 9B | Ogre Hill | 2741 | Greenheugh Patch | Mastcam | MR Mcam14367 |
| 9C | Enlarged area of 9A | 2692 | Greenheugh Patch | Mastcam | MR Mcam13955 |
| 9D | Enlarged area of 9A | 2692 | Greenheugh Patch | Mastcam | ML Mcam14085 |
| 9F | Hilltop Extension | 2729 | Greenheugh Patch | Mastcam | MR Mcam14303 |
| 10A | Machir Bay | 2699 | Greenheugh Patch | Mastcam | MR Mcam14125 |
| 10B | Enlarged area in 10A | 2699 | Greenheugh Patch | Mastcam | MR Mcam14125 |
| 10C | Enlarged area in 10A | 2699 | Greenheugh Patch | Mastcam | MR Mcam14125 |
